# Improvements and inter-laboratory implementation and optimization of blood-based single-locus age prediction models using DNA methylation of the *ELOVL2* promoter

**DOI:** 10.1038/s41598-020-72567-6

**Published:** 2020-09-24

**Authors:** Imene Garali, Mourad Sahbatou, Antoine Daunay, Laura G. Baudrin, Victor Renault, Yosra Bouyacoub, Jean-François Deleuze, Alexandre How-Kit

**Affiliations:** 1 Laboratory for Bioinformatics, Foundation Jean Dausset-CEPH, Paris, France; 2 Laboratory of Excellence GenMed, Paris, France; 3 Laboratory for Human Genetics, Foundation Jean Dausset-CEPH, Paris, France; 4 Laboratory for Genomics, Foundation Jean Dausset-CEPH, 75010 Paris, France; 5 Centre National de Recherche en Génomique Humaine, CEA, Institut François Jacob, Evry, France

**Keywords:** Genetics, Epigenetics, Biological techniques, Epigenetics analysis, Molecular biology, Epigenetics

## Abstract

Several blood-based age prediction models have been developed using less than a dozen to more than a hundred DNA methylation biomarkers. Only one model (Z-P1) based on pyrosequencing has been developed using DNA methylation of a single locus located in the *ELOVL2* promoter, which is considered as one of the best age-prediction biomarker. Although multi-locus models generally present better performances compared to the single-locus model, they require more DNA and present more inter-laboratory variations impacting the predictions. Here we developed 17,018 single-locus age prediction models based on DNA methylation of the *ELOVL2* promoter from pooled data of four different studies (training set of 1,028 individuals aged from 0 and 91 years) using six different statistical approaches and testing every combination of the 7 CpGs, aiming to improve the prediction performances and reduce the effects of inter-laboratory variations. Compared to Z-P1 model, three statistical models with the optimal combinations of CpGs presented improved performances (MAD of 4.41–4.77 in the testing set of 385 individuals) and no age-dependent bias. In an independent testing set of 100 individuals (19–65 years), we showed that the prediction accuracy could be further improved by using different CpG combinations and increasing the number of technical replicates (MAD of 4.17).

## Introduction

Aging is a complex biological process influenced by both genetic and environmental factors and characterized by the progressive decline of several physiological, cellular and molecular functions^1,2^. Several studies have aimed to identify potential biological and/or molecular biomarkers of aging correlating with chronological age and to use them to develop age prediction models^3,4^. Four types of DNA-based biomarkers of aging have been identified among the molecular biomarkers: telomere length^3,5,6^, mitochondria mutations^6,7^, signal joint T-cell receptor rearrangement excision circles and DNA methylation^8–11^.

To date, DNA methylation is considered as the most promising molecular biomarker for age prediction and several DNA methylation-based biomarkers of aging correlating with chronological age have therefore been used for the construction of prediction models to estimate the chronological age of individuals, which could be particularly useful in forensic science and for public health concerns^12^. In forensics, the ability to precisely determine the chronological age of samples from DNA methylation-based age prediction models could greatly help investigators to identify and find unknown individuals^13^. In other bio-medical applications, the estimated age from DNA methylation could give an estimation of the biological age^4^ and could also be an indicator of different diseases, risks and health conditions when compared to the chronological age of individuals^14–17^.

There are numerous DNA methylation biomarkers, i.e. CpG sites, whose methylation status correlates to chronological age similarly in each individual, defined as the ‘epigenetic clock’^12,18^. These biomarkers have been used to develop several DNA methylation-based age-prediction models that are based either on a high number of CpGs requiring the use of genome-wide epigenotyping array technologies^19,20^ or a lower number of CpGs using locus-specific technologies such as pyrosequencing^21–23^. *ELOVL fatty acid elongase 2* (*ELOVL2*) has shown to be one of the best DNA methylation biomarkers correlating with the chronological age of individuals among the age-prediction biomarkers and has therefore been included in several age prediction models^21,24^. The models based upon DNA methylation analysis by pyrosequencing mainly use blood as a source of DNA and present the advantage of requiring only a small number of analyzed CpGs (down to 2 CpGs) and a minimal amount of DNA, which is particularly useful for forensic applications^21^.

To our knowledge only one blood-based age prediction model has been developed from a single locus located in the *ELOVL2* promoter and used multiple linear regression^25^, while all the other models were developed as multi-locus models from at least two different loci^21^. In a recent study, we evaluated and inter-compared six-age prediction models on a cohort of 100 individuals aged from 19 to 65 years^26^, including a single-locus model (Zbiec-Piekarska 1^25^) and five multi-locus models (Bekaert^27^, Park^28^, Thong^29^, Weidner^30^, and Zbiec-Piekarska 2^31^). The models presenting the best age prediction accuracy were the multi-locus models of Bekaert and Thong (MAD of 4.5–5.2 years and SEE of 6.8–7.2 years) followed by the single-locus model of Zbiec-Piekarska 1 (MAD of 6.8 year and SEE of 8.6 years) while the models presenting the worst age prediction performances (MAD of 7.2–8.7 years and SEE of 9.2–10.3 years) were the three other multi-locus models of Weidner, Park and Zbiec-Piekarska 2^26^. The latter MAD were much higher than the ones described in their original studies, and we suggested that these differences could be principally attributed to inter-laboratory variations during the implementation of the different pyrosequencing assays^26^. Thus, the use of several loci and pyrosequencing assays might increase the variability in the predicted age estimates of the models when run in different laboratories.

In the present study, we aimed to develop improved blood-based single-locus age prediction models using *ELOVL2* promoter methylation evaluating every combination of CpGs and different statistical models. We also aimed to propose a simple approach for the implementation and optimization of the age-prediction models across laboratories that could limit the effect of inter-laboratory variations on age predictions. To set up our models, we used freely available DNA methylation data from 1,413 individuals aged between 0 and 91 years taken from four independent previously published studies^27,28,31,32^, which were divided into a training set (1,028 individuals) and a testing set (385 individuals). Seven CpG sites were considered inside the *ELOVL2* promoter and we used multiple quadratic regression and three machine learning approaches, namely support vector machine, gradient boosting regressor and missMDA, to identify the CpG combinations with the best age prediction accuracy. The performances of our models were also compared to those of the already published single-locus model^25^ on the same data set and we further evaluated the different approaches on a second independent set of 100 individuals. To further improve the age prediction accuracy, we also evaluated the possibility to estimate the age of the samples using the age averages of the different models and/or of different types of technical replicate experiments that would be easy to setup in other laboratories.

## Material and methods

### Description of the publicly available data sets and comparison of *ELOVL2* promoter methylation data from four independent studies

As increasing the number of individuals included in training sets improved the age prediction accuracy^33,34^, we searched for different previously published pyrosequencing datasets of *ELOVL2* promoter DNA methylation. Four datasets comprising 206^27^, 420^31^, 765^28^ and 100^32^ blood samples from individuals aged between 0 and 91 years (Supplementary Table 1) were identified. Park et al.^28^ and Zbiec-Piekarska et al.^31^ used the same pyrosequencing assays and Bekaert et al.^27^ and Cho et al.^32^ used two other slightly different pyrosequencing assays (Supplementary Table 2). The data of the 1,491 samples presented similar DNA methylation values according to the age of individuals with the exception of CpG7 in the Park et al. study^28^, where 73 samples presented lower DNA methylation values that could be considered as outliers (Supplementary Fig. 1). Thus, the 73 samples from this study as well as five samples from Cho et al. study presenting missing values were excluded from our subsequent analyses. The seven CpG sites all presented strong positive correlation (r > 0.70) indicating that they could all be good estimators of the chronological age (Table 1). The 1,413 samples were randomly divided in a training and testing sets including 1,028 and 385 individuals, respectively.

**Table 1:** Correlation between chronological age and DNA methylation for the seven CpGs analyzed located in the *ELOVL2* promoter.

CpG	Chromosome location (GRCh38)	Bekaert^27^ (n = 206)	Zbiec-Piekarska^31^ (n = 420)	Park^28^ (n = 692)	Cho^32^ (n = 95)	All (n = 1414)
1	Chr6: 11,044,661	0.898	0.837	0.940	0.860	0.904
2	Chr6: 11,044,655	0.915	0.799	0.920	0.834	0.884
3	Chr6: 11,044,647	0.866	0.803	0.897	0.818	0.852
4	Chr6: 11,044,644	0.912	0.841	0.902	0.872	0.851
5	Chr6: 11,044,642	0.924	0.881	0.906	0.871	0.893
6	Chr6: 11,044,640	0.939	0.877	0.935	0.821	0.911
7	Chr6: 11,044,634	0.876	0.910	0.907	0.887	0.878

### Description of the independent testing set

We used an independent testing set of 100 blood samples from individuals aged between 19 and 65 years, which were used in our previously published study^26^. We used *ELOVL2* PCR and pyrosequencing assays published from the Zbiec-Pierkarska study^25^, which presented a slight PCR bias in favor of unmethylated DNA with a polynomial fit curve on DNA methylation standards (Supplementary Fig. 2). For each sample, 1 µg of DNA was used for bisulfite treatment followed by three PCR reactions and two subsequent pyrosequencing experiments (PSQ) per PCR (Supplementary Fig. 3A). Correlation analysis of DNA methylation showed that two pyrosequencing replicates from the same PCR reaction showed a better correlation (A1:A2, B1:B2 and C1:C2) than from two different PCR reactions (A1/2:B1/2, A1/2:C1/2 and B1/2:C1/2, Supplementary Fig. 3B), which might influence the age prediction accuracy. Thus, we considered DNA methylation obtained from five types of replicate experiments: one replicate (1 PCR and 1 PSQ per PCR), two replicates (1 PCR and 2 PSQ per PCR or 2 PCR and 1 PSQ per PCR), three replicates (3 PCR and 1 PSQ per PCR) and six replicates (3 PCR and 2 PSQ per PCR).

### Human blood DNA samples

The study was conducted in accordance with current ethical and legal frameworks. All methods were performed in accordance to the recommendations of the French National Committee of Ethics (Comité Consultatif National d’Ethique pour les Sciences de la Vie et de la Santé). Anonymized blood samples were obtained after informed consent from healthy donors through the French blood bank, EFS (Etablissement Français du Sang, Paris, France-research agreement 15/EFS/012). Peripheral blood samples were derived from 100 healthy French donors aged between 19 and 65 years (Supplementary Table 1). Buffy coats were obtained from blood after 10 min of centrifugation at 1,600*g* and frozen at − 80 °C before DNA extraction. DNA extraction was performed on buffy coats using the QIAmp DNA blood mini Kit (Qiagen) on a QIAcube robotic workstation (Qiagen) according to the manufacturer’s instructions. DNA quantification was performed using the Qubit dsDNA HS assay Kit on a Qubit 3 Fluorometer (Thermo Fischer Scientific) according to the manufacturer’s instructions. These DNA samples were already analysed in our previous study^26^ and were used to perform a new sodium bisulfite treatment in the present study.

### Bisulfite conversion and bisulfite treated DNA quantification

Bisulfite conversion of DNA was performed on 1 µg of genomic DNA, using the EpiTect Bisulfite Kit 48 (Qiagen) on a QIAcube robotic workstation (Qiagen) according to the manufacturer’s instructions. Bisulfite-treated DNA was quantified using the quantitative real-time PCR QC1 methylight assay^35^ and diluted to a final concentration of 20 ng/µl for DNA methylation analysis by pyrosequencing.

### PCR amplification

*ELOVL2* promoter region was amplified as described in^26^. 20 µL PCR reactions was performed in a Mastercyler Pro S (Eppendorf) with 20 ng of bisulfite-treated DNA as a template. The PCR mix included 1 × HotStar Taq DNA polymerase buffer, 1.8 mM of additional MgCl_2_, 200 µM of each dNTP, 200 nM of each primer (ELOVL2_F: Biotin-AGGGGAGTAGGGTAAGTGAGG and ELOVL2_R: AACAAAACCATTTCCCCCTAATAT) and 2 U of HotStar Taq DNA polymerase. Cycling conditions included an initial denaturation step performed for 10 min at 95 °C, followed by 50 cycles of 30 s denaturation at 95 °C, 30 s annealing at 60 °C and 30 s elongation at 72 °C. The final step included 5 min elongation at 72 °C.

### DNA methylation analysis by pyrosequencing

10 µL of PCR product was purified and prepared for pyrosequencing (sequencing oligo ELOVL2_Seq: ACAACCAATAAATATTCCTAAAACT and pyrosequencing analysis sequence: CCR_1_TGAAACR_2_TTGAAGACCR_3_CCR_4_CR_5_CR_6_AAACCR_7_AC) according to a previously described protocol^36,37^. DNA methylation analysis was performed using the PyroMark Gold SQA Q96 Kit (Qiagen) on a PyroMark Q96 MD (Qiagen) and analyzed with PyroMark CpG software (Qiagen).

### Statistical analysis and graphical representation

All statistical analysis and graphical representations were performed using R (https://www.r-project.org/) or MS Excel (Microsoft). We developed the age prediction models using multiple quadratic regression (MQR), support vector machine (SVM), gradient boosting regressor (GBR) and MissMDA (mMDA) by testing every combination of the 7 CpG sites to improve the estimations of predicted ages. MQR was performed for each of the 7 CpG sites by considering the methylation value for each sample and their squares so that in total 14 variables were used. For the MQR, SVM and GBR approaches, we split our data into a training set and testing set. We fit our model on the training set and made predictions on the testing set. For mMDA, the value to predict (the age of individuals) was considered as a missing value and the data were not split into training and testing data. mMDA used a single dataset with non-missing and missing values corresponding to training set data with non-missing ages and testing set data with missing ages, respectively. mMDA imputed the missing ages with PCA taking into account the similarities between the observations and the relationships between variables. For convenience, data with known age and those with missing values were named “testing set” and “training set” in the rest of our manuscript, respectively. For the support vector machine, we tested Linear (SVM_l_), Polynomial (SVM_p_) and Radial kernel (SVM_r_). For the GBR we used decision trees with a different number of iterations. For each age prediction model, the accuracy of age prediction was evaluated by the mean absolute deviation (MAD) and the root mean square error (RMSE) and the correlation analyses were assessed using the Pearson R correlation coefficient.

## Results

### Development and evaluation of the performances of *ELOVL2* single-locus age prediction models

The previously developed *ELOVL2* Zbiec-Piekarska model was based on multiple linear regression using CpGs 5 and 7^25^. We tested six different statistical approaches for the development of *ELOVL2* single-locus age prediction models. We evaluated multiple quadratic regression (MQR), as some CpGs from *ELOVL2* were shown to present a better correlation with the chronological age using a quadratic rather than a linear regression model^27^, support-vector machines with radial (SVM_r_), linear (SVM_l_) and polynomial (SVM_p_) functions, the latter function presenting the best age prediction accuracy in a study using DNA methylation of 12 multi-locus CpG sites obtained by NGS that evaluated 17 statistical models^38^, gradient boosting regressor (GBR) that presented the best age prediction accuracy in a 6 loci age-prediction model using epigenotyping microarray DNA methylation data^39^ and missMDA (mMDA)^40,41^, which has never been used to date in an age prediction model. A previous study showed that age-related DNA methylation changes were logarithmic^42^, however, we did not include this function in our regression models, as the relationship between chronological age and DNA methylation of *ELOVL2* was better fitted in our data by a linear or quadratic regression for most CpGs (Supplementary Table 3). We also evaluated the correlation between DNA methylation of the seven CpGs. Our analysis showed that the CpGs were highly correlated with each other (Supplementary Fig. 4), suggesting that multicollinearity could be present in our models. However, we decided not to take this parameter into account and not to correct for it in the development of our models, as we only focused on predictions that should not be affected by multicollinearity^43^. Thus, we used every combination of 1–7 CpGs sites corresponding to 127 possible combinations for each statistical model to evaluate the age prediction performances, except for the multiple quadratic regression approach where we considered 14 variables corresponding to DNA methylation values and their squared counterparts for the 7 CpG sites, resulting in 16,383 possible combinations. Thus, 17,018 age prediction models were developed in our study.

**Figure 1:**
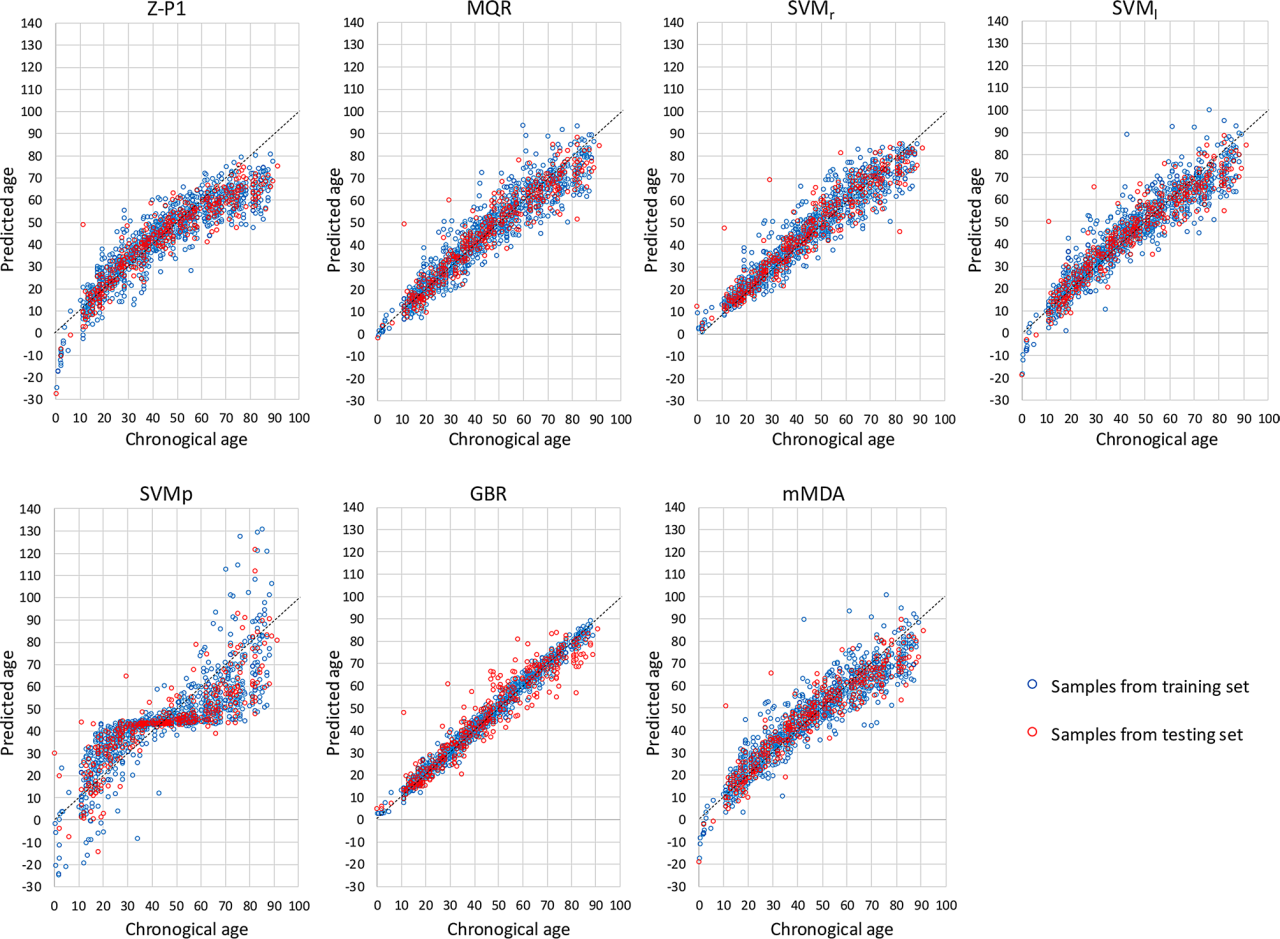
Scatterplots of predicted age and chronological age of the training and testing samples obtained with *ELOVL2* age-prediction models based on six different statistical approaches. The plotted data were obtained from the combination of CpGs giving the best age prediction accuracy on the training set. Z-P1, Zbiec-Piekarska model^25^ using multiple linear regression; MQR, multiple quadratic regression; SVM, support vector machine with radial kernel (_r_), linear (_l_) and polynomial (_p_) functions; GBR, gradient boosting regressor; mMDA, missMDA. Four out-of-scale values (y-axis) are missing for SVMp.

We calculated the Pearson R coefficient, MAD and RMSE for the 17,018 age prediction models based on the six different statistical approaches, which have been summarized in Supplementary Fig. 5. The results first showed that for each tested model, the combination of CpGs giving the best age prediction accuracy slightly differed according to the data set taken as reference (training or testing set), where fewer CpGs were required to obtain the best age prediction accuracies when using the testing set as the reference set (Table 2). We could also note that the highest difference observed for the age prediction accuracy between the training and validation set was for GBR (MAD of 1.99–2.38 for the training set and MAD of 4.43–5.55 for the testing set) and that mMDA presented the least number of CpGs (three) for the best age prediction accuracy (Fig. 1 and Table 2). Our results also showed that five out of the six tested models (MQR, SVM_r_, SVM_l_, BGR and mMDA) presented better age prediction performances compared to those obtained with the multiple linear regression model of Zbiec-Pierkarska (Z-P1) in both the training and validation sets (Fig. 1, Table 2 and Supplementary Fig. 6) or the validation set of the original study (MAD of 5.75)^25^. This suggests that these different statistical models were more accurate for age prediction from *ELOVL2* than multiple linear regression, notably for the samples from the youngest and oldest individuals whose predicted age were under-evaluated in the models of Zbiec-Pierkarska (Fig. 1 and Supplementary Fig. 6). In each model tested, we observed one sample of the testing set with a chronological age of 11 years that systematically presented an over-estimation of its predicted age (> 40 years). In its original study, this sample also presented an age of 50 years predicted from a multiple linear regression model based on 3 CpGs located in *ELOVL2*, *ZNF423* and *CCDC102B*^28^. These results suggest that this sample could come from an older individual.

**Table 2:** Age prediction performances of the different statistical models on the training and testing sets.

Model	Best performance from Training (T)/Testing (V) sets^a^	Number of CpGs	CpG combination	Training set			Testing set		
				R	MAD	RMSE	R	MAD	RMSE
Zbiec-Pierkarska 1	–	2	CpG_5,7_	0.918	6.885	9.127	0.932	6.397	8.803
MQR	T	9	CpG_1–2 & 4–6_ & CpG_2_^2^_,4_^2^_,6_^2^_–7_^2^	0.945	5.133	6.975	0.950	4.773	6.730
	V	8	CpG_4–6_ & CpG_2_^2^_–4_^2^_,6_^2^_–7_^2^	0.941	5.229	7.184	0.953	4.574	6.559
SVM_r_	T	6	CpG_1–3,5–7_	0.956	4.555	6.229	0.953	4.464	6.544
	V	5	CpG_2–3,5–7_	0.9546	4.6139	6.3257	0.9534	4.4101	6.4919
SVM_l_	T	7	CpG_1–7_	0.935	5.575	7.531	0.943	5.221	7.194
	V	5	CpG_2–6_	0.930	5.650	7.793	0.945	5.130	7.058
SVM_p_	T	7	CpG_1–7_	0.799	9.946	13.046	0.830	9.734	12.124
	V	5	CpG_3–7_	0.778	10.456	13.582	0.833	9.465	12.098
GBR	T	7	CpG_1–7_	0.992	1.993	2.627	0.953	4.549	6.520
	V	5	CpG_2,4–7_	0.989	2.378	3.121	0.955	4.426	6.398
mMDA	T	3	CpG_1,5–6_	0.933	5.650	7.625	0.940	5.320	7.357
	V	3	CpG_2,5–6_	0.929	5.801	7.855	0.943	5.231	7.223

^a^For each statistical model, both CpG combinations giving the best age prediction accuracy according to the training (T) and testing (V) sets were included in the table.

Thus, the models giving the best age prediction accuracy on the testing set were in order SVM_r_, GBR and MQR (MAD of 4.41–4.77 and RMSE of 6.40–6.73) followed by SVM_l_ and mMDA (MAD of 5.13–5.32 and RMSE of 7.06–7.36), while SVM_p_ presented the poorest age prediction accuracy (MAD of 9.47–9.73 and RMSE of 12.10–12.12) and was omitted for all other downstream analyses (Fig. 1, Table 2 and Supplementary Fig. 6). We also evaluated whether averaging the predicted age between the different statistical models used could further improve the age prediction accuracy. Our results showed that multiple model averaging from *ELOVL2* DNA methylation could slightly improve the age prediction accuracy (MAD of 4.36 and RMSE of 6.36 from the averaging of GBR, SVM_r_ and MQR predictions, Supplementary Table 4).

### Improvement of age-prediction accuracy of the models by increasing the number of technical replicates and inter-laboratory implementation and optimization of the models

We further evaluated the different models and the impact of the increase of technical replicates on age prediction accuracy using an independent testing set of 100 blood samples from individuals aged between 19 and 65 years (see description in the “Material and methods”). We first evaluated the Z-P1, MQR, SVM_r_, SVM_l_, BGR and mMDA models on the dataset composed of one technical replicate. The results showed that for each statistical model the combinations of CpGs giving the best age prediction accuracy in this independent testing set required a lower number of CpGs (1–4 CpGs) than previously identified in the training and testing sets and relied mainly on CpGs 6 and 7 (Table 3). Thus, the combinations of CpGs previously identified in Table 2 with each statistical model presented lower age prediction performances in this independent dataset than those obtained with these new CpG combinations (Supplementary Table 5). This indicated that some inter-laboratory variations might influence the combinations of CpGs giving the best performance for age prediction. These variations could be observed in our independent testing set for CpGs 1–3, whose average DNA methylation was slightly higher than that of the initial training and testing sets (Supplementary Fig. 7) and that could thereby explain their absence in the CpG combinations giving the best prediction accuracy (Table 3).

**Table 3:** Age prediction performances of the different statistical models on an independent validation set.

Model	Number of CpGs	CpGs	Estimators	Training set (n = 1,028)	Testing set 1 (n = 385)	Independent testing set 2 (n = 100)				
						1 PCR and 1 PSQ/PCR (1 replicate)	1 PCR and 2 PSQ/PCR (2 replicates)	2 PCR and 1 PSQ/PCR (2 replicates)	3 PCR and 1 PSQ/PCR (3 replicates)	3 PCR and 2 PSQ/PCR (6 replicates)
Zbiec-Pierkarska 1	2	CpG_5,7_	R	0.918	0.932	0.880	0.893	0.902	0.909	0.914
			MAD	6.885	6.397	5.445	5.319	5.147	5.050	5.011
			RMSE	9.127	8.803	6.870	6.624	6.440	6.290	6.201
MQR	4	CpG_6_ & CpG_4_^2^_,6_^2^_–7_^2^	R	0.934	0.945	0.904	0.911	0.919	0.924	0.927
			MAD	5.521	4.910	4.786	4.619	4.425	4.266	4.232
			RMSE	7.574	7.057	6.225	5.996	5.765	5.598	5.504
SVMr	2	CpG_6,7_	R	0.947	0.948	0.902	0.906	0.917	0.923	0.925
			MAD	5.051	4.701	4.784	4.668	4.388	4.211	4.174
			RMSE	6.843	6.833	6.287	6.140	5.771	5.581	5.515
SVMl	2	CpG_6,7_	R	0.905	0.927	0.902	0.905	0.917	0.922	0.923
			MAD	6.246	6.036	5.536	5.484	5.289	5.211	5.197
			RMSE	9.078	8.095	6.874	6.796	6.525	6.404	6.375
BGR	2	CpG_6,7_	R	0.976	0.947	0.900	0.904	0.913	0.919	0.920
			MAD	3.471	4.772	4.892	4.842	4.577	4.436	4.469
			RMSE	4.660	6.931	6.397	6.314	5.973	5.803	5.741
mMDA	1	CpG_6_	R	0.906	0.927	0.902	0.905	0.917	0.922	0.923
			MAD	6.291	6.079	5.926	5.875	5.736	5.673	5.598
			RMSE	9.008	8.104	7.234	7.158	6.932	6.826	6.772

**Figure 2:**
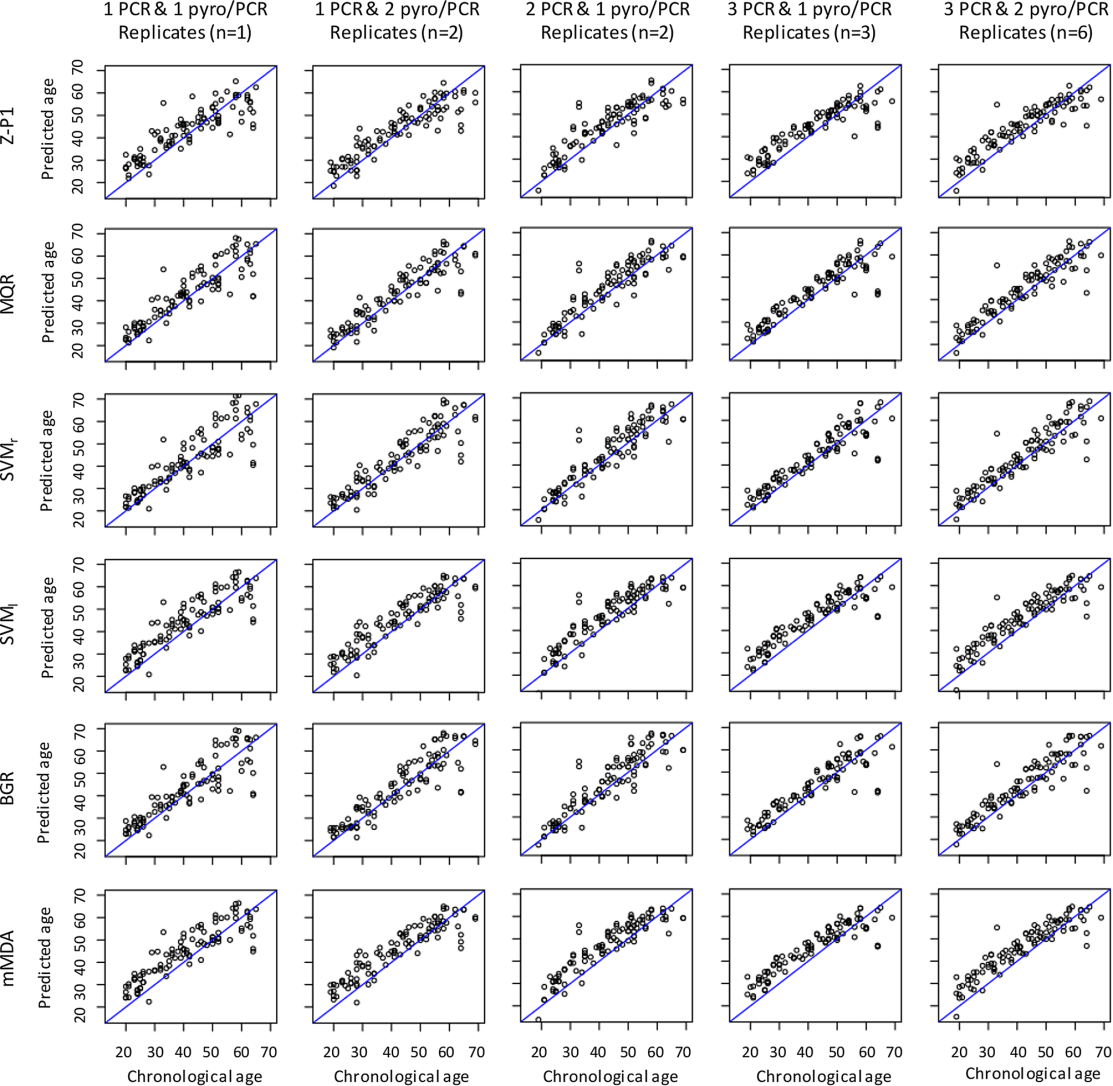
Scatterplots of predicted age and chronological age of the independent testing set of 100 blood samples from individuals of 19–65 years obtained with *ELOVL2* age-prediction models based on six different statistical approaches. The plotted data were obtained from the combination of CpGs giving the best age prediction accuracy on this independent testing set. Due to replicate measures per sample and to allow comparison between conditions, only one age prediction value per sample was randomly picked for representation. Z-P1, Zbiec-Piekarska model^25^ using multiple linear regression; MQR, multiple quadratic regression; SVM, support vector machine with radial kernel (_r_) and linear (_l_) functions; GBR, gradient boosting regressor; mMDA, missMDA.

The statistical models presenting the best age prediction accuracy in this independent testing set were MQR, SVM_r_ and BGR (MAD of 4.78–4.89 and RMSE of 6.23–6.40) followed by Z-P1, SVM_l_, and mMDA, which presented slightly lower performance (MAD of 5.45–5.93 and RMSE of 6.87–7.23, Fig. 2, Table 3 and Supplementary Fig. 8). Regarding the effect of technical replicates, our results showed that the performances of each tested model were improved as the number of technical replicates increased, where duplicating PCR reactions improved age prediction more than duplicating pyrosequencing experiments from a single PCR (Fig. 2, Table 3 and Supplementary Fig. 8). The best performances were achieved with MQR and SVM_r_ from six replicates experiments (MAD of 4.17–4.23 and RMSE of 5.50–5.52, Table 3). As previously shown, averaging the predicted age between the different statistical models could further improve the age prediction accuracy (MAD of 4.156 and RMSE of 5.461 using the averaging of SVM_r_ and MQR predictions and the six-replicate dataset, Supplementary Table 6).

## Discussion

Several multi-locus age prediction models based on DNA methylation of blood or other body fluids relied on less than a dozen markers using mainly pyrosequencing to several tens or hundreds of loci using epigenotyping arrays^12,21,26^. To our knowledge, only a single-locus model based on two CpGs of the *ELOVL2* promoter and multiple linear regression has been proposed to date, in which the MAD of age predictions were of 5.03 and 5.75 in the training (303 samples) and testing (124 samples) sets respectively^25^. Despite multi-locus age prediction models generally presented better age prediction accuracy than the *ELOVL2* single-locus model in their original studies, our recent study evaluating six blood based age prediction models using DNA methylation analysis by pyrosequencing showed that the performances of multi-locus models could sometimes be poorer in independent validation studies^26^. This could be attributed to inter-laboratory variations and discrepancies resulting from slight experimental differences accumulated during the different stages of sample processing, which could potentially increase as the number of PCR and pyrosequencing assays increases^26^. In a more recent study Pfeifer et al. evaluated two published multi-locus age prediction models using an independent validation set^44^. Their results presented worse age prediction performances (MAD of 9.84 instead of 3.75 in the original study^27^) that they also attributed to inter-laboratory variations caused by some differences in experimental conditions (reagents used, PCR and pyrosequencing conditions and devices…)^44^.

The objective of the present study was thereby to improve the age prediction performances of single-locus blood-based age prediction models using *ELOVL2* promoter DNA methylation and also to propose an approach for the implementation and optimization of the best models in different laboratories in order to deal with the effects of inter-laboratory variations that could decrease the age-prediction performances^26,44^. Using three different parameters: (1) the choice of the statistical model, (2) the combination of CpG sites and (3) technical replications, we aimed to improve the age prediction that would avoid the need to increase the number of analyzed loci and to use multi-locus models, thus greatly simplifying the experimental procedures, the costs and also the amount of DNA required. Combining DNA methylation data of the *ELOVL2* promoter from four independent studies allowed us to take into account some inter-laboratory variations in the developed models while increasing the training set sample size, which should result in an improved precision of age estimates^33,34^. The use of different PCR and pyrosequencing assays in these different studies could partially explain the observed inter-laboratory variations in the DNA methylation data. Our results showed that for the best combinations of CpGs obtained with the six tested statistical models (MQR, SVM_r_, SVM_l_, SVM_p_, BGR and mMDA), five presented better age prediction accuracy compared to the Zbiec-Piekarska model in our training and testing sets (Fig. 1 and Table 2), while only three statistical models (MQR, SVM_r_ and BGR) outperformed the same model when compared to the performances of its original study^25^. We also showed that averaging the predictions of these three models could be a way to improve the age prediction accuracy slightly (Supplementary Table 4). It should be highlighted that 127 (multiple) linear regression models (from every combination of the 7 CpGs) were also tested in our study in MQR and their age prediction performances were among the worst 30% (not shown), confirming that the relationship between age and *ELOVL2* DNA methylation is better modeled using multiple quadratic regression^27^. Moreover, Zbiec-Piekarska model under-evaluated the age of the youngest and oldest individuals in our study and this tendency was already visible in the original study^25^. Of note, the SVM_p_ showed the poorest age prediction performances in our study although it had been identified as the best approach for age estimations among 17 statistical models including also SVM_r_ in a recent study using 12 different loci^38^. This suggests that the selected markers and/or the number of markers used could greatly influence the age prediction accuracy of the statistical model.

In an independent test set of 100 blood samples, we evaluated MQR, SVM_r_, SVM_l_, BGR and mMDA on their age prediction accuracy as well as the effect of technical replicates of PCR and pyrosequencing experiments generated in our laboratory. Our results showed that the best age prediction accuracy was always obtained with different combinations of fewer CpG sites than previously identified (Tables 2 and 3). This indicates that the inter-laboratory variations occurring during the implementation of pyrosequencing assays due to some differences in the experimental conditions might influence the age prediction accuracy, as was also shown in the multi-locus models^26^. As a consequence, the best combination of CpGs could also vary across laboratories and should therefore be systematically evaluated to obtain the best age prediction performances. We have also evaluated the effect of replicate PCR and/or pyrosequencing experiments on age prediction accuracy in this independent validation cohort, which has been rarely performed in other studies. We showed that increasing PCR and pyrosequencing replicates could be a simple way to improve the age prediction accuracy in each tested model, with a stronger effect of pyrosequencing replicates from independent PCR reactions than from the same PCR reaction (Fig. 2, Table 3 and Supplementary Fig. 7). The best age prediction performance obtained with SVM_r_ (MAD of 4.17, Table 3) was even better than the performances from the best multi-locus model of Bekaert identified in our previous study that compared five multilocus-models (MAD of 4.5^26^).

Due to the constraints inherent in our study, we used different combinations of CpGs of the *ELOVL2* promoter that are very close in the DNA sequence. We showed that their DNA methylations were highly correlated, which could have introduced multicollinearity in our developed models. For example, multicollinearity could be detected in the three MQR equations presented in our study for variables with variance inflation factors (VIF) higher than 10, thus inducing less confident estimations of their coefficients in the equations (Supplementary Table 7). However, although multicollinearity is an issue for explanatory modeling, it is not the case when we are only interested in predictions as in the context of our study^43^. Nevertheless, in order to handle collinear variables in the models, ridge regression (RR), principal component regression (PCR) or partial least squares regression (PLS) could have been performed. A principal components analysis describing variance in our dataset could also be first performed and used to reduce the number of correlated variables (Supplementary Fig. 9).

Our study showed that the use of a single-locus blood-based age prediction model could achieve improved performances equaling multi-locus models. For optimal inter-laboratory implementation and age prediction performances of *ELOVL2* single-locus age prediction models, we recommend the use of our experimental conditions for *ELOVL2* PCR and pyrosequencing assays combined with one of the three best statistical models identified in our study: SVM_r_, MQR or GBR. The evaluation of the selected statistical models trained on our provided training dataset using every combination of the 7 CpG sites (14 variables for MQR) should systematically be performed on an independent set of testing samples (obtained from individuals with as large an age difference as possible). It would allow the identification of the best CpG combination that should be used in the different laboratories to obtain the best estimates of predicted age. Two or three measures of *ELOVL2* DNA methylation from independent PCR experiments should then be used to further improve the age prediction accuracy of the samples of interest. Another approach has also been proposed for inter-laboratory adaptation of multi-locus DNA methylation-based age prediction models in order to manage and deal with inter-laboratory variations that decreased the age prediction performances^44^. It required retraining the models using an independent training set, in addition to an independent validation set^44^. Our proposed approach could be simpler, faster and less expensive as it only requires an independent validation set.

In conclusion, we showed that the performances of a single-locus age-prediction model based on *ELOVL2* promoter methylation could be improved by modifying the statistical model used, the combination of CpGs chosen and also the number of technical replicates. With these improvements, the *ELOVL2* single-locus model could therefore match the performances of multi-locus models while greatly simplifying the experimental procedures, the costs and also the amount of DNA needed due to the need of only one locus, which could be particularly useful for forensic applications. The development of single-locus age prediction models based on *ELOVL2* promoter methylation was also particularly interesting as DNA methylation of this age-prediction biomarker, contrary to other DNA methylation-based age-prediction biomarkers, has proven to be correlated with age in most types of tissues^45^ and could thereby potentially be used on different types of samples without requiring many changes. Our model could also potentially be used to study the modification of the epigenetic clock in individuals with different health conditions, as shown in numerous studies using high-throughput multi-locus age prediction models relying on epigenotyping microarray data^12^ and low-throughput multi-locus age prediction models based on pyrosequencing^17,46^. Further evaluations of our single-locus age prediction models based on *ELOVL2* promoter methylation should be performed on samples from different types of tissues as well as from individuals with different health conditions and/or diseases to define the applicability of these models to such samples.

## Supplementary information

Supplementary Information 1.

Supplementary Information 2.

Supplementary Dataset 1.

Supplementary Dataset 2.

Supplementary Dataset 3.

Supplementary Dataset 4.

Supplementary Dataset 5.

## Data Availability

The training set was provided as Supplementary Datasets 1 (for MQR) and 2 (for SVM_r_ and GBR) and the testing set was provided as Supplementary Datatsets 3 (for MQR) and 4 (for SVM_r_ and GBR). A customizable list of variables (CpGs) is also required for MQR and is provided as Supplementary Dataset 5.

## Code availability

The codes were provided for the three best statistical models identified in our study, i.e. MQR, SVMr and GBR (Supplementary Information 2) and allowed users to identify the CpG combinations giving the best age prediction performances with their own testing set.
