# Supplementary material for: Improvements and inter-laboratory implementation and optimization of blood-based single-locus age prediction models using DNA methylation of the ELOVL2 promoter
Source: Sci Rep. 2020 Sep 24;10:15652. doi: 10.1038/s41598-020-72567-6 (PMC7515898; doi:10.1038/s41598-020-72567-6)
Supplement: Supplementary file 1 — Supplementary Information 1. [file 41598_2020_72567_MOESM1_ESM.pdf]

Supplementary Information for:

Improvements and inter-laboratory implementation and optimization of blood-based single-locus age prediction models using DNA methylation of the *ELOVL2* promoter

Imène Garali[1,2*], Mourad Sahbatou[3*], Antoine Daunay[4], Laura G. Baudrin[2,4], Victor Renault[1], Yosra Bouyacoub[2,4], Jean-François Deleuze[1-5] & Alexandre How-Kit[3‡]

[1] Laboratory for Bioinformatics, Foundation Jean Dausset – CEPH, Paris, France

[2] Laboratory of Excellence GenMed, Paris, France

[3] Laboratory for Human Genetics, Foundation Jean Dausset – CEPH, Paris, France

[4] Laboratory for Genomics, Foundation Jean Dausset – CEPH, Paris, France

[5] Centre National de Recherche en Génomique Humaine, CEA, Institut François Jacob, Evry, France

[*] Both authors contributed equally to this work.

[‡] ***Correspondence to:***

Alexandre How-Kit, Ph.D., Laboratory for Genomics, Foundation Jean Dausset - CEPH, Paris, F-75010, France, Tel.: +33-(0)1- 53725146, email: alexandre.how-kit@fjd-ceph.org

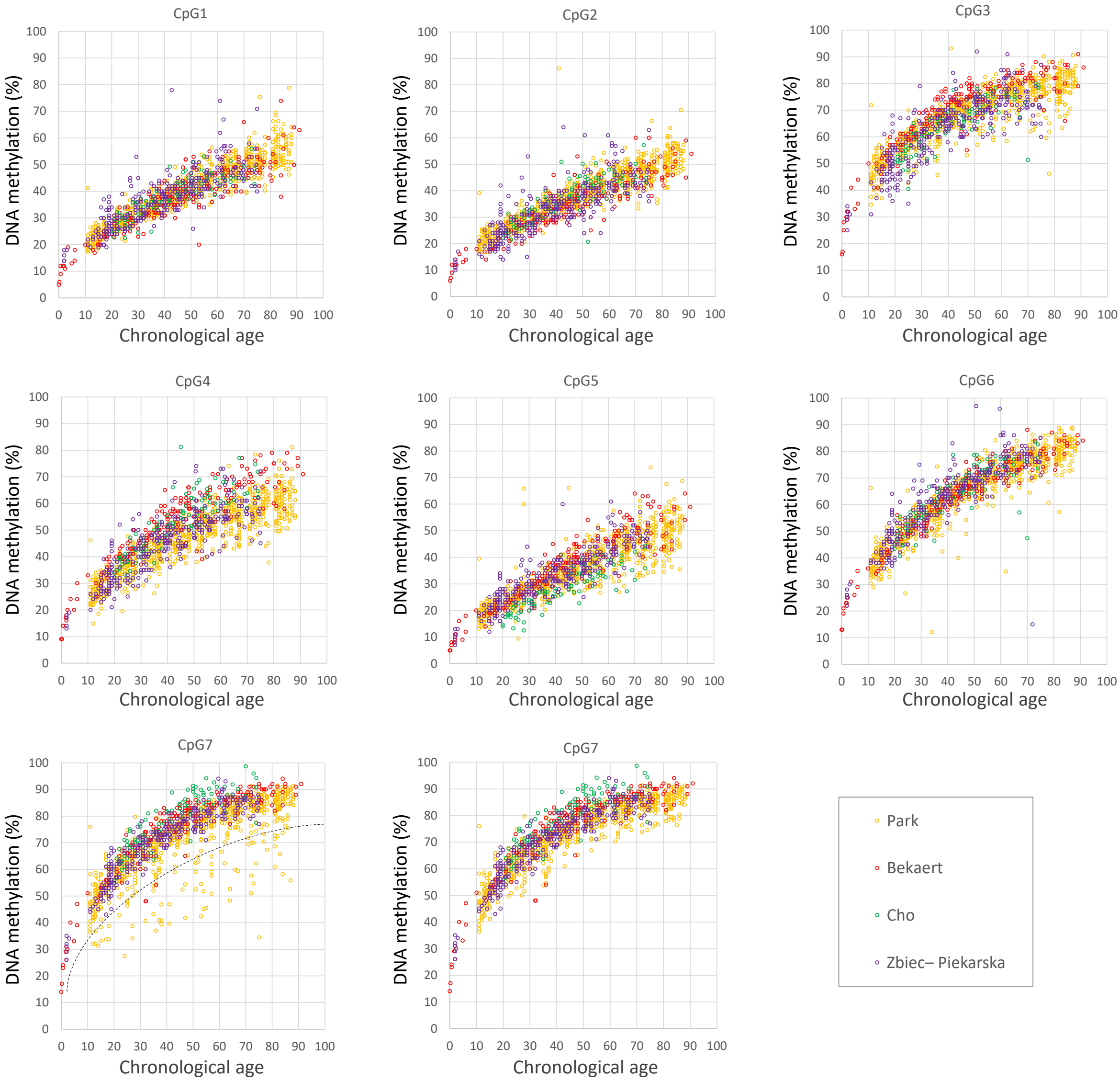

Supplementary Figure 1. Variation of the DNA methylation of the 7 CpGs located in the *ELOVL2* promoter in the blood samples from the four previously published studies.

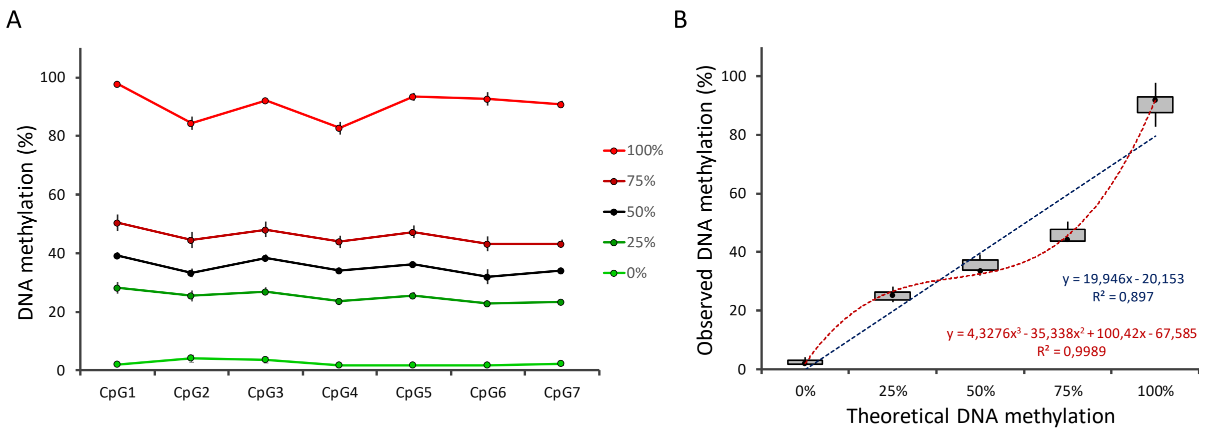

Supplementary Figure 2. *ELOVL2* DNA methylation pyrosequencing assay linearity assessment. **A**. DNA methylation patterns of the 7 analyzed CpGs in *ELOVL2* promoter using 0%, 25%, 50%, 75% and 100% DNA methylation standards (the 0% and 100% DNA methylation standards were purchased from Qiagen and mixed in 3:1, 1:1 and 1:3 equimolar ratios to obtain 25%, 50% and 75% DNA methylation standards). **B.** Boxplot of the expected and obtained DNA methylation values for the 7 analyzed CpGs in the *ELOVL2* promoter. The linear and polynomial regression curves and their corresponding equations and coefficients of determination R^2 are indicated. Experiments were performed in triplicate and vertical bars in the line graphs represent standard deviation.

A

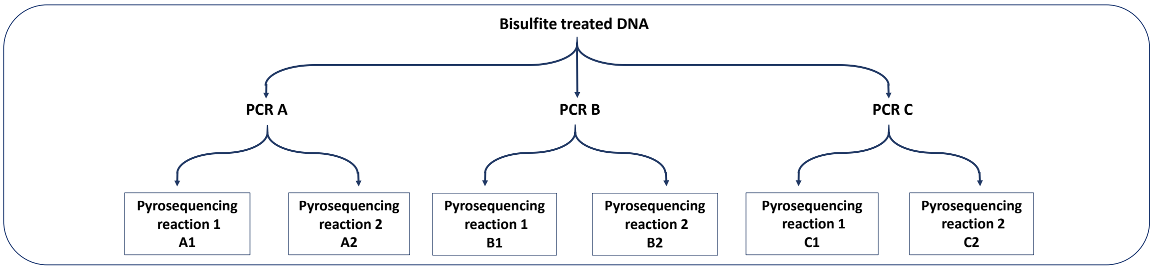

B

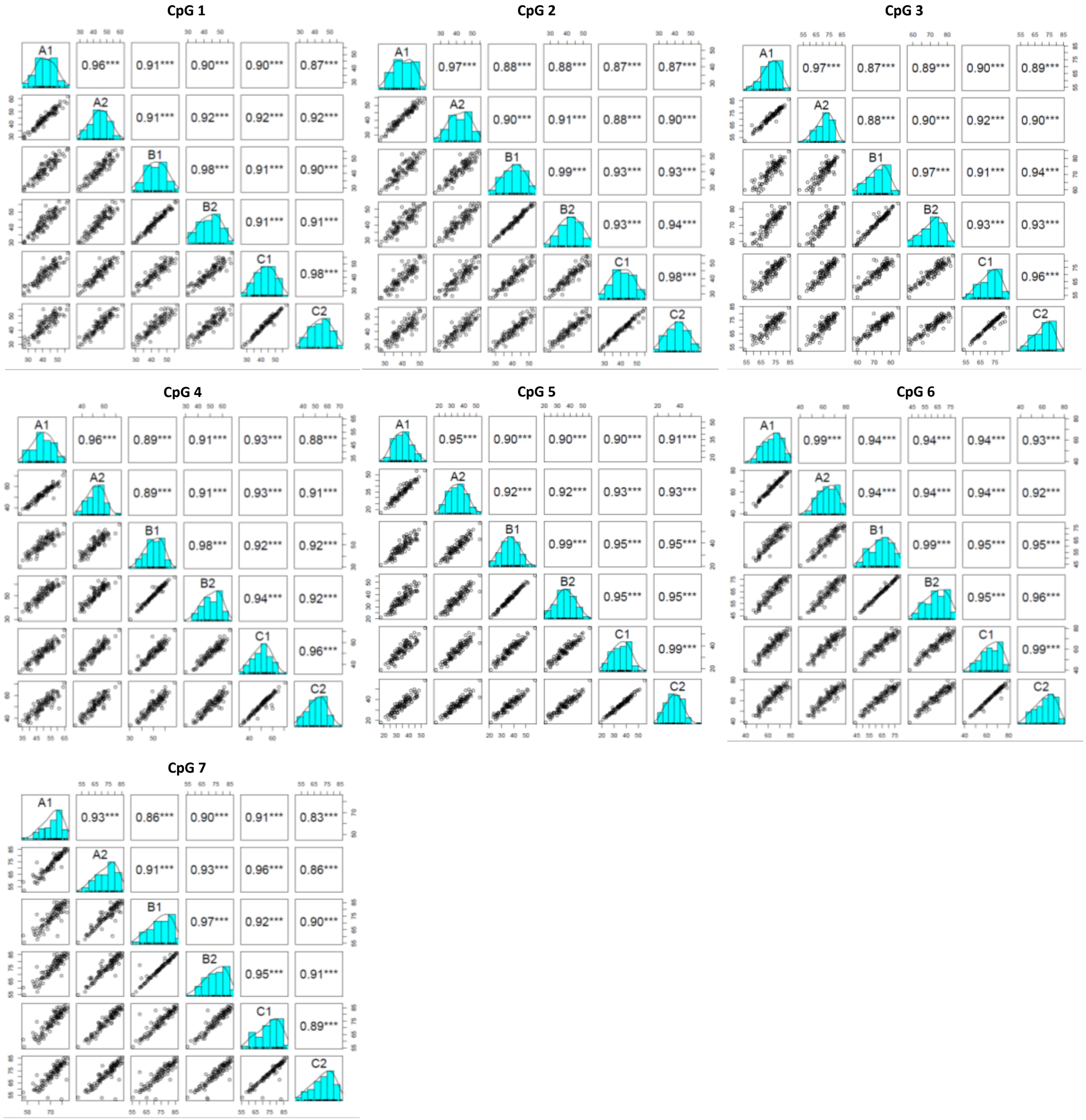

Supplementary figure 3. Effect of PCR and pyrosequencing replicate experiments on *ELOVL2* promoter DNA methylation of the independent testing set (100 samples). **A**. Description of the experimental workflow. **B**. Correlation plots of DNA methylation values of each CpG obtained between every PCR and pyrosequencing replicate. The Pearson R coefficients and scatterplots between two replicates (A1, A2, B1, B2, C1 and C2) are given for each CpG. *** indicates p-value < 0.001 for the correlation tests.

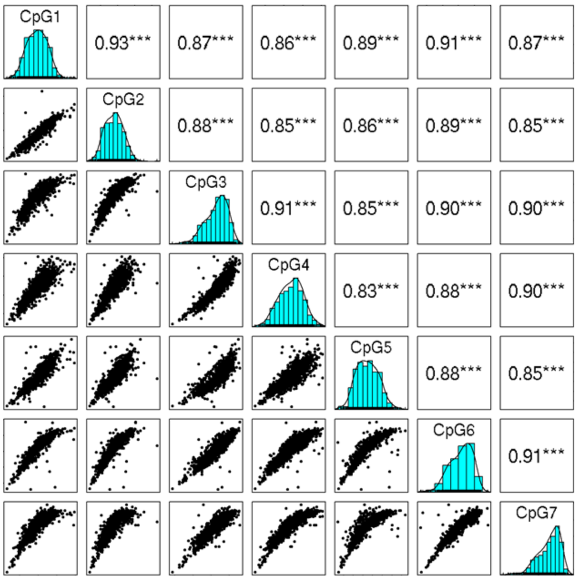

Supplementary Figure 4. Correlation matrix of DNA methylation of the seven CpGs from the *ELOVL2* promoter used in our study using the training set. *** indicates *p*-value < 0.001

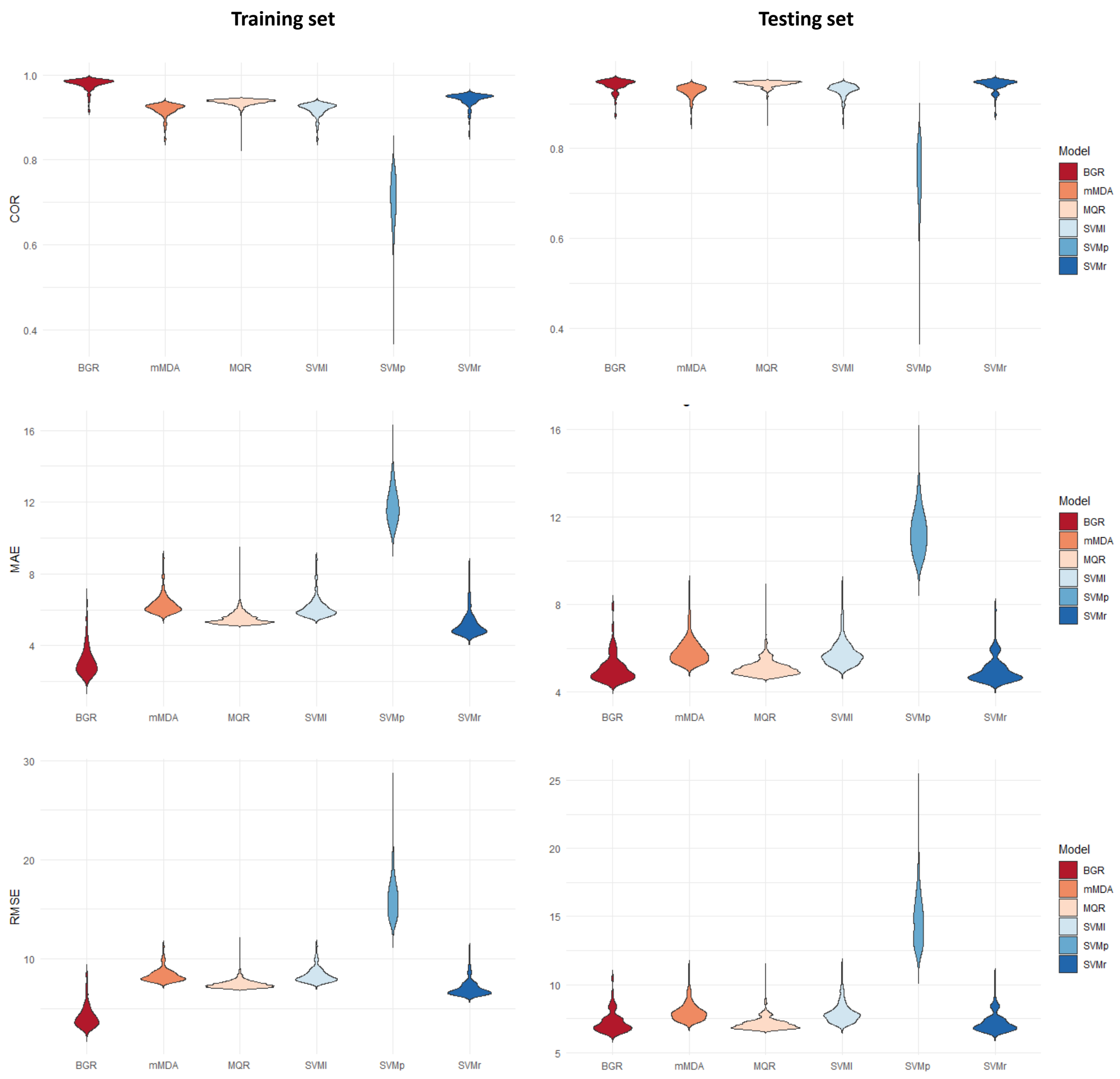

Supplementary Figure 5. Violin plots showing the distribution of Pearson correlation coefficients (CORR), mean absolute deviations (MAD) and root mean square errors (RMSE) obtained with the training and testing sets for the 17 018 age prediction models based on DNA methylation of the *ELOVL2* promoter. For each indicator, the values were grouped according to the statistical approach used.

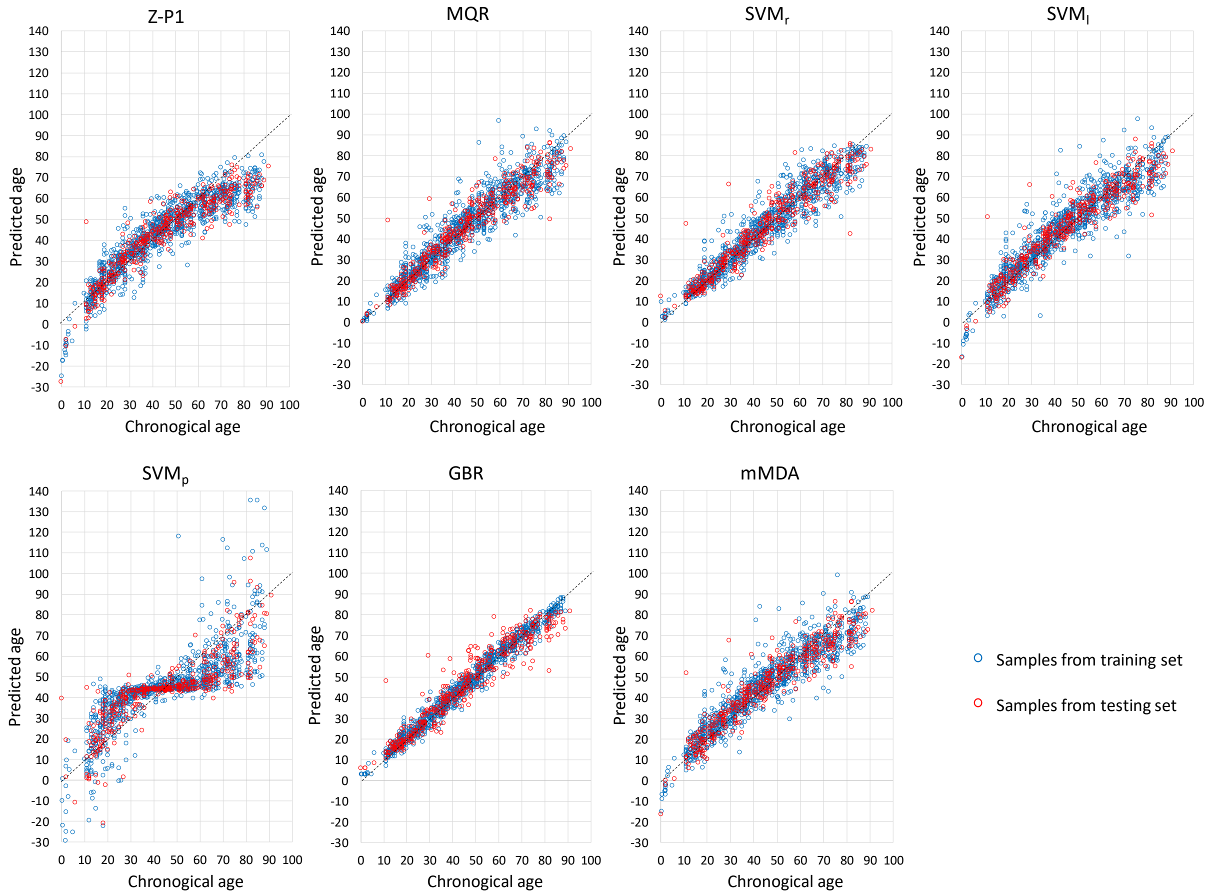

Supplementary Figure 6. Scatterplots of predicted age and chronological age of the training and testing samples obtained with *ELOVL2* age-prediction models based on seven different statistical approaches. The plotted data were obtained from the combination of CpGs giving the best age prediction accuracy on the testing set. Z-P1, Zbiec-Piekarska model [1] using multiple linear regression; MQR, multiple quadratic regression; SVM, support vector machine with radial kernel ($_r$), linear ($_l$) and polynomial ($_p$) functions; GBR, gradient boosting regressor; mMDA, missMDA. Four out-of-scale values (y-axis) are missing for SVMp.

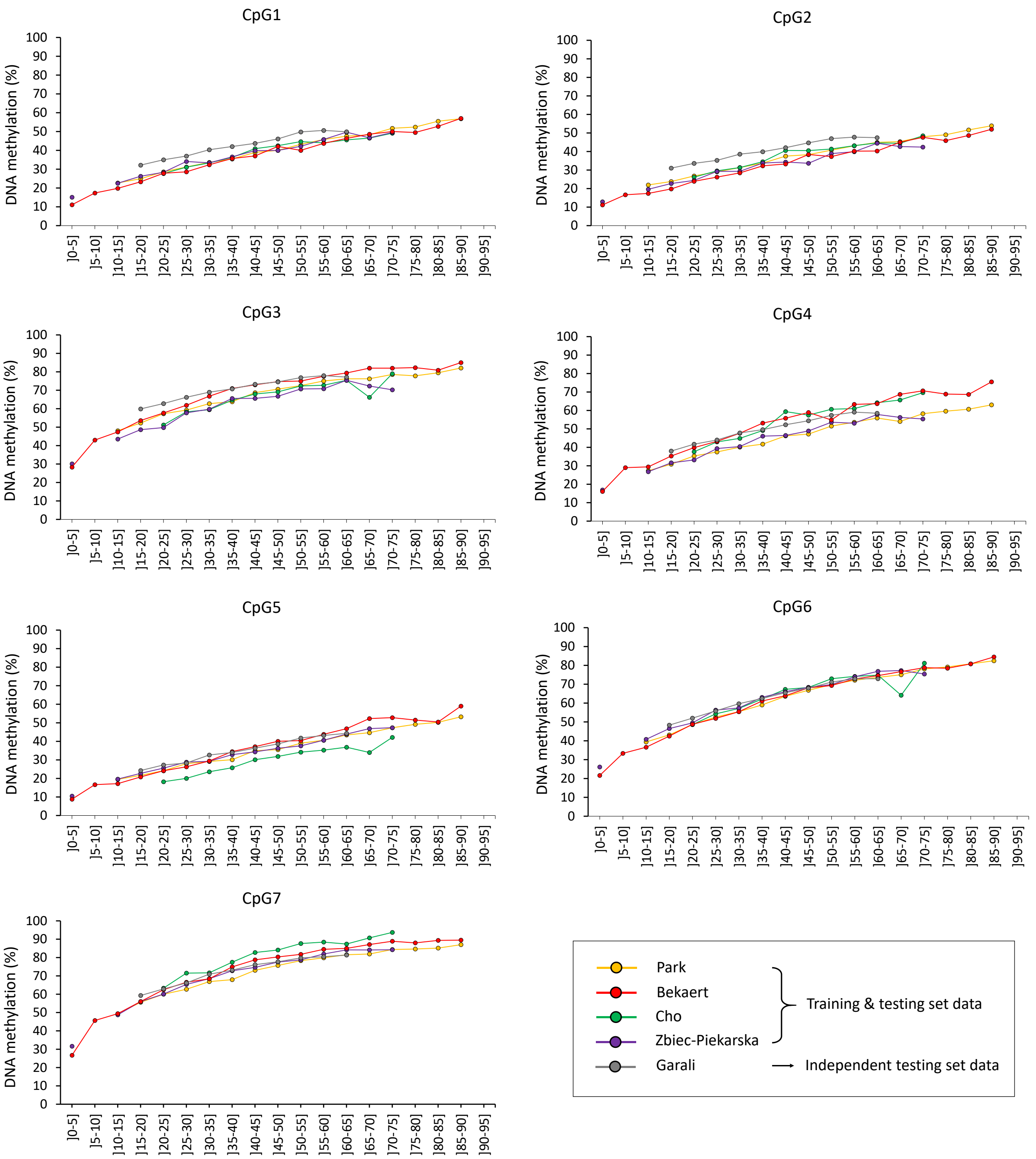

Supplementary Figure 7. Average DNA methylation of the 7 CpGs located in the *ELOVL2* promoter from the blood samples of the four previously published studies (Park [2], Bekaert [3], Cho [4] and Zbiec-Pierkarska [5]) and our independent testing set according to age groups in 5 year increments. At least 2 individuals are present in each age group.

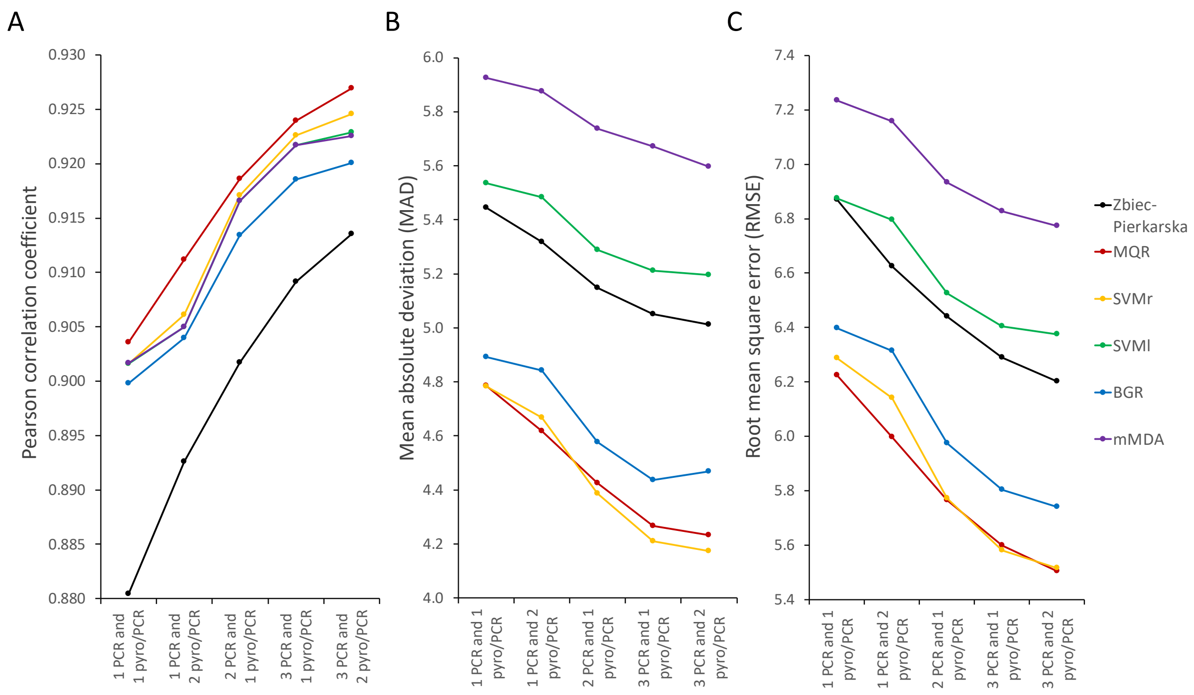

Supplementary Figure 8. Effect of PCR and/or pyrosequencing replicate experiments on age prediction performances in an independent testing set of 100 blood samples. For each tested statistical model, the Pearson R correlation coefficient (**A**), the mean absolute deviation (**B**) and the root-mean-square error (**C**) of the predicted and chronological ages are shown according to the increased number of PCR and/or pyrosequencing replicates. The estimators are given for the combinations of CpGs giving the best age prediction performances (see Table 3).

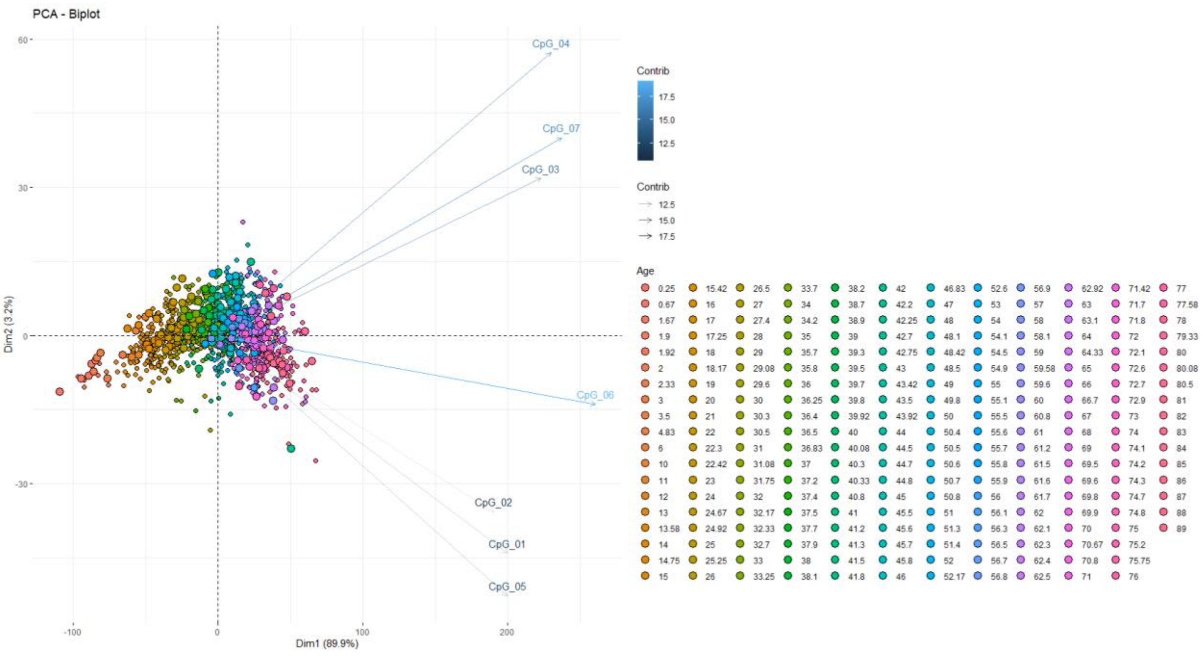

Supplementary Figure 9. Principal component analysis (PCA) biplot of the first two principal component coefficients, observations and observed variables using the training dataset (1028 individuals). The PCA biplot shows both PC scores of samples (dots) and loadings of variables (vectors). The further away these vectors are from a PC origin, the more influence they have on that PC. Loading plots also hint at how variables correlate with one another.

Supplementary Table 1. Description of the samples and dataset used

Study	Blood Samples[a]		Training Set[b]		Testing Set (I)[b]		Independent Testing Set (II)[c]	
	N	Age range	N	Age range	N	Age range	N	Age range
Bekaert [3]	206	0-91	150	0-89	56	0-75	-	-
Zbiec-Piekarska [5]	420	2-75	299	2-75	121	2-75	-	-
Park [2]	765	11-90	507	11-88	185	11-88	-	-
Cho [4]	100	20-74	72	20-74	23	22-74	-	-
Daunay [6] & Garali	-	-	-	-	-	-	100	19-65
Total	1491	0-91	1028	0-91	385	0-88	100	19-65

[a] The total number of samples from the original studies are indicated, regardless of the original training/testing sets.
[b] New training and testing sets used in our study and randomly generated.
[c] Independent validation set from the same samples analyzed in [6] but totally reprocessed in our study.

Supplementary Table 2. List of PCR and pyrosequencing primers used in the different studies

Study	Forward PCR Primer	Reverse PCR Primer	Pyrosequencing Primer	Sequence to Analyse
Zbiec-Piekarska et al., 2015	Biotin-GGGGAGTAGGGTAAGTGAGG	AACAAAACCATTTCCCCCTAATAT	ACAACCAATAAATATTCCTAAAACT	$CCR_1TGAAACR_2TTGAAGACCR_3CCR_4CR_5CR_6AAACCR_7AC$
Bekaert et al., 2015	Biotin-AGGGGYGTAGGGTAAGTGAG	AAACCCAACTATAAACAAAACCAA	AATAAATATTCCTAAAACTCC	$R_1TAAACR_2TTAAACCR_3CCR_4CR_5CR_6AAACCR_7ACRCCRACTAAACCTA$
Park et al., 2016	Biotin-GGGGAGTAGGGTAAGTGAGG	AACAAAACCATTTCCCCCTAATAT	ACAACCAATAAATATTCCTAAAACT	$CCR_1TGAAACR_2TTGAAGACCR_3CCR_4CR_5CR_6AAACCR_7AC$
Cho et al., 2017	AGGGGAGTAGGGTAAGTGAGG	Biotin-AACCATTTCCCCCTAATATATACTTCA	GGGAGGAGATTTGTAGGTTT	$AGTYGGYGTY_7GGTTTY_6GY_5GY_4GGY_3GGTTTAAY_2GTTTAY_1GGA$
Garali et al., 2020	Biotin-GGGGAGTAGGGTAAGTGAGG	AACAAAACCATTTCCCCCTAATAT	ACAACCAATAAATATTCCTAAAACT	$CCR_1TGAAACR_2TTGAAGACCR_3CCR_4CR_5CR_6AAACCR_7AC$

Supplementary Table 3. Comparison of linear, quadratic and exponential regressions of the chronological age of the training set on the DNA methylation of each CpG in the *ELOVL2* promoter.

CpG	Chromosome location (GRCh38)	Linear					Quadratic					Exponential				
		R^2	*p*-value	Corr. Coeff. R	MAD	RMSE	R^2	*p*-value	Corr. Coeff. R	MAD	RMSE	R^2	*p*-value	Corr. Coeff. R	MAD	RMSE
**1**	Chr6: 11,044,661	**0.821**	<2,2e-16	**0.900**	**6.806**	**9.425**	0.766	<2,2e-16	0.884	7.606	10.201	n,a	<2e-16	0.872	8.840	11.141
**2**	Chr6: 11,044,655	**0.774**	<2,2e-16	**0.897**	**7.284**	**9.595**	0.707	<2,2e-16	0.885	7.941	10.296	n,a	<2e-16	0.881	9.256	11.373
**3**	Chr6: 11,044,647	0.724	<2,2e-16	0.856	8.796	11.171	**0.743**	<2,2e-16	**0.867**	**8.207**	**10.748**	n,a	<2e-16	0.861	8.353	11.033
**4**	Chr6: 11,044,644	**0.716**	<2,2e-16	**0.865**	**8.379**	**10.891**	0.680	<2,2e-16	0.854	8.839	11.380	n,a	<2e-16	0.844	9.613	12.037
**5**	Chr6: 11,044,642	**0.781**	<2,2e-16	**0.918**	**6.545**	**8.690**	0.723	<2,2e-16	0.899	7.492	9.679	n,a	<2e-16	0.894	8.233	10.383
**6**	Chr6: 11,044,640	0.820	<2,2e-16	0.927	6.094	8.114	**0.850**	<2,2e-16	**0.941**	**5.141**	**7.315**	n,a	<2e-16	0.941	5.461	7.523
**7**	Chr6: 11,044,634	0.764	<2,2e-16	0.887	7.702	9.955	0.794	<2,2e-16	0.907	6.821	9.085	n,a	<2e-16	**0.910**	**6.479**	**8.979**

The estimators of the three types of regressions giving the best performances have been bolded.

Supplementary Table 4. Age prediction performances obtained for the testing set by averaging the ages predicted with the different statistical models tested.

Methods Combinations [1,2]	R	MAD	RMSE
GBR + SVMr + MQR	0.955	4.364	6.363
GBR + SVMr	0.955	4.368	6.371
GBR + MQR	0.955	4.397	6.389
**SVMr**	**0.953**	**4.410**	**6.492**
SVMr + MQR	0.955	4.419	6.420
**GBR**	**0.955**	**4.426**	**6.398**
GBR + SVMl + SVMr + MQR	0.955	4.446	6.426
GBR + SVMl + SVMr	0.954	4.452	6.439
GBR + SVMr + mMDA + MQR	0.954	4.462	6.446
GBR + SVMr + mMDA	0.954	4.479	6.473
GBR + SVMl + MQR	0.954	4.531	6.480
SVMl + SVMr + MQR	0.953	4.547	6.512
GBR + mMDA + MQR	0.954	4.557	6.509
GBR + SVMl + SVMr + mMDA + MQR	0.953	4.559	6.516
SVMr + mMDA + MQR	0.953	4.568	6.541
**MQR**	**0.953**	**4.574**	**6.559**
GBR + SVMl	0.953	4.582	6.523
GBR + SVMl + SVMr + mMDA	0.953	4.595	6.560
SVMl + SVMr	0.952	4.611	6.585
GBR + mMDA	0.953	4.622	6.584
SVMr + mMDA	0.952	4.652	6.648
GBR + SVMl + mMDA + MQR	0.952	4.664	6.598
SVMl + SVMr + mMDA + MQR	0.952	4.679	6.626
GBR + SVMl + mMDA	0.951	4.751	6.693
SVMl + MQR	0.951	4.762	6.676
SVMl + SVMr + mMDA	0.950	4.779	6.739
mMDA + MQR	0.951	4.791	6.725
SVMl + mMDA + MQR	0.949	4.882	6.805
**SVMl**	**0.945**	**5.130**	**7.058**
SVMl + mMDA	0.944	5.167	7.119
**mMDA**	**0.943**	**5.245**	**7.233**

[1] for each statistical model, the ages were predicted using the combination of CpG giving the best age prediction performance according to the testing (V) set (see Table 2)

[2] age prediction performances of each statistical approach alone are indicated in bold.

Supplementary Table 5. Age prediction performances of the different statistical models on the independent testing set using the same CpG combinations as defined in Table 2.

Model	Best age-prediction performance from Training (T)/Testing (V) sets[1]	Number of CpGs	CpG combination	Independent Testing set (1 replicates: 1 PCR and 1 PSQ/PCR)		
				R	MAD	RMSE
Zbiec-Pierkarska 1	-	2	$CpG_{5, 7}$	0.880	5.445	6.870
MQR	T	9	$CpG_{1\text{-}2\ \&\ 4\text{-}6}$ & $CpG_{2^2, 4^2, 6^2\text{-}7^2}$	0.912	6.602	7.712
	V	8	$CpG_{4\text{-}6}$ & $CpG_{2^2\text{-}4^2, 6^2\text{-}7^2}$	0.908	5.667	6.901
SVMr	T	6	$CpG_{1\text{-}3, 5\text{-}7}$	0.906	6.666	7.950
	V	5	$CpG_{2\text{-}3, 5\text{-}7}$	0.901	6.128	7.478
SVMl	T	7	$CpG_{1\text{-}7}$	0.908	7.911	9.041
	V	5	$CpG_{2\text{-}6}$	0.906	7.406	8.581
BGR	T	7	$CpG_{1\text{-}7}$	0.899	6.727	7.864
	V	5	$CpG_{2, 4\text{-}7}$	0.898	6.177	7.407
mMDA	T	3	$CpG_{1, 5\text{-}6}$	0.905	7.973	9.094
	V	3	$CpG_{2, 5\text{-}6}$	0.905	8.172	9.308

[1] For each statistical model, both CpG combinations giving the best age prediction accuracy according to the training (T) and testing (V) sets were included in the table.

Supplementary Table 6. Age prediction performances obtained for the independent testing set by averaging the ages predicted with the different statistical models tested.

Method combination [1]	1 PCR and 1 PSQ/PCR (1 replicate)			3 PCR and 2 PSQ/PCR (6 replicates)		
	R	MAD	RMSE	R	MAD	RMSE
MQR + SVMr	0.905	4.717	6.189	0.927	4.156	5.461
MQR + SVMr + GBR	0.904	4.753	6.234	0.925	4.256	5.532
**SVMr**	**0.902**	**4.784**	**6.287**	**0.925**	**4.174**	**5.515**
**MQR**	**0.904**	**4.786**	**6.225**	**0.927**	**4.232**	**5.504**
MQR + GBR	0.903	4.797	6.267	0.925	4.316	5.582
SVMr + GBR	0.902	4.798	6.296	0.923	4.311	5.593
MQR + SVMr + SVMl + GBR	0.905	4.889	6.293	0.926	4.436	5.658
**GBR**	**0.899**	**4.892**	**6.397**	**0.920**	**4.469**	**5.741**
MQR + SVMr + SVMl	0.906	4.927	6.307	0.927	4.434	5.671
MQR + SVMr + GBR + mMDA	0.905	4.966	6.350	0.926	4.524	5.729
SVMr + SVMl + GBR	0.905	4.966	6.356	0.925	4.528	5.731
MQR + SVMl + GBR	0.906	4.971	6.346	0.926	4.539	5.756
MQR + SVMr + mMDA	0.906	5.030	6.386	0.927	4.556	5.771
MQR + SVMr + SVMl + GBR + mMDA	0.906	5.058	6.413	0.926	4.623	5.825
SVMr + SVMl	0.904	5.062	6.429	0.926	4.573	5.794
SVMr + GBR + mMDA	0.905	5.067	6.434	0.925	4.644	5.829
MQR + GBR + mMDA	0.906	5.082	6.433	0.926	4.665	5.862
MQR + SVMl	0.906	5.091	6.422	0.927	4.658	5.856
SVMl + GBR	0.905	5.116	6.468	0.925	4.728	5.921
MQR + SVMr + SVMl + mMDA	0.906	5.134	6.475	0.927	4.698	5.896
SVMr + SVMl + GBR + mMDA	0.905	5.157	6.503	0.926	4.743	5.930
MQR + SVMl + GBR + mMDA	0.906	5.174	6.504	0.926	4.775	5.964
SVMr + mMDA	0.904	5.212	6.557	0.926	4.766	5.954
MQR + mMDA	0.906	5.262	6.568	0.927	4.859	6.034
GBR + mMDA	0.905	5.295	6.612	0.925	4.920	6.095
SVMr + SVMl + mMDA	0.904	5.299	6.641	0.926	4.885	6.074
MQR + SVMl + mMDA	0.905	5.337	6.649	0.926	4.953	6.137
SVMl + GBR + mMDA	0.905	5.353	6.670	0.925	4.989	6.166
**SVMl**	**0.902**	**5.536**	**6.874**	**0.923**	**5.197**	**6.375**
SVMl + mMDA	0.902	5.728	7.049	0.923	5.422	6.583
**mMDA**	**0.902**	**5.936**	**7.240**	**0.923**	**5.654**	**6.806**

[1] age prediction performances of each statistical approach alone are indicated in bold.

Supplementary Table 7. Detection of multicollinearity in the three MQR models presented in Tables 2 and 3.

Regression equation[1]	*p*-value	Variance Inflation Factor (VIF) analysis		
		Variables	Tolerance	VIF
Predicted age = 0.960664 + 0.423366 x CpG_1 + 0.621481 x CpG_2 + 0.22014 x CpG_4 + 0.275027 x CpG_5 - 1.098227 x CpG_6 - 0.006766 x CpG_2^2 - 0.002802 x CpG_4^2 + 0.013366 x CpG_6^2 + 0.002782 x CpG_7^2	< 2.2e-16	1 CpG_1	0.092740254	10.782804
		2 CpG_2	0.012957728	77.174025
		3 CpG_4	0.009571836	104.473169
		4 CpG_5	0.162888166	6.139181
		5 CpG_6	0.009365895	106.770364
		6 CpG_2^2	0.01799795	55.561883
		7 CpG_4^2	0.012631093	79.169715
		8 CpG_6^2	0.009072519	110.222973
		9 CpG_7^2	0.102189798	9.785713
Predicted age = 2.6012533 + 0.5762165 x CpG_4 + 0.3983775 x CpG_5 - 0.9083127 x CpG_6 + 0.00235 x CpG_2^2 - 0.0002596 x CpG_3^2 - 0.0057862 x CpG_4^2 + 0.0126432 x CpG_6^2 + 0.0032594 x CpG_7^2	< 2.2e-16	1 CpG_4	0.009778364	102.266595
		2 CpG_5	0.17607202	5.679494
		3 CpG_6	0.010169183	98.336313
		4 CpG_2^2	0.196257787	5.095339
		5 CpG_3^2	0.107450535	9.306608
		6 CpG_4^2	0.013652849	73.244786
		7 CpG_6^2	0.009591711	104.256689
		8 CpG_7^2	0.104977366	9.525863
Predicted age = 13.4944951 - 0.8224263 x CpG_6 - 0.0001978 x CpG_4^2 + 0.0143482 x CpG_6^2 + 0.0044380 x CpG_7^2	< 2.2e-16	1 CpG_6	0.01931145	51.782739
		2 CpG_4^2	0.20245162	4.939452
		3 CpG_6^2	0.01943566	51.451811
		4 CpG_7^2	0.12580673	7.9487

[1] The equations use DNA methylation values expressed in percentage.

References

1 Zbiec-Piekarska, R. *et al.* Examination of DNA methylation status of the ELOVL2 marker may be useful for human age prediction in forensic science. *Forensic Sci Int Genet* 14, 161-167, doi:10.1016/j.fsigen.2014.10.002 (2015).

2 Park, J. L. *et al.* Identification and evaluation of age-correlated DNA methylation markers for forensic use. *Forensic Sci Int Genet* 23, 64-70, doi:10.1016/j.fsigen.2016.03.005 (2016).

3 Bekaert, B., Kamalandua, A., Zapico, S. C., Van de Voorde, W. & Decorte, R. Improved age determination of blood and teeth samples using a selected set of DNA methylation markers. *Epigenetics* 10, 922-930, doi:10.1080/15592294.2015.1080413 (2015).

4 Cho, S. *et al.* Independent validation of DNA-based approaches for age prediction in blood. *Forensic Sci Int Genet* 29, 250-256, doi:10.1016/j.fsigen.2017.04.020 (2017).

5 Zbiec-Piekarska, R. *et al.* Development of a forensically useful age prediction method based on DNA methylation analysis. *Forensic Sci Int Genet* 17, 173-179, doi:10.1016/j.fsigen.2015.05.001 (2015).

6 Daunay, A., Baudrin, L. G., Deleuze, J. F. & How-Kit, A. Evaluation of six blood-based age prediction models using DNA methylation analysis by pyrosequencing. *Sci Rep* 9, 8862, doi:10.1038/s41598-019-45197-w (2019).
