# Supplementary material for: Improvements and inter-laboratory implementation and optimization of blood-based single-locus age prediction models using DNA methylation of the ELOVL2 promoter
Source: Sci Rep. 2020 Sep 24;10:15652. doi: 10.1038/s41598-020-72567-6 (PMC7515898; doi:10.1038/s41598-020-72567-6)
Supplement: Supplementary file 2 — Supplementary Information 2. [file 41598_2020_72567_MOESM2_ESM.docx]

Practical session for:

Improvements and inter-laboratory implementation and optimization of blood-based single-locus age prediction models using DNA methylation of the *ELOVL2* promoter

Imène Garali^1,2*^, Mourad Sahbatou^3*^, Antoine Daunay^4^, Laura G. Baudrin^2,4^, Victor Renault^1^, Yosra Bouyacoub^2,4^, Jean-François Deleuze^1-5^ & Alexandre How-Kit^3ǂ^

^1^ Laboratory for Bioinformatics, Foundation Jean Dausset – CEPH, Paris, France

^2^ Laboratory of Excellence GenMed, Paris, France

^3^ Laboratory for Human Genetics, Foundation Jean Dausset – CEPH, Paris, France

^4^ Laboratory for Genomics, Foundation Jean Dausset – CEPH, Paris, France

^5^ Centre National de Recherche en Génomique Humaine, CEA, Institut François Jacob, Evry, France

^*^ Both authors contributed equally to this work.

^ǂ^ ***Correspondence to:***

Alexandre How-Kit, Ph.D., Laboratory for Genomics, Foundation Jean Dausset - CEPH, Paris, F-75010, France, Tel.: +33-(0)1- 53725146, email: [alexandre.how-kit@fjd-ceph.org](mailto:alexandre.how-kit@fjd-ceph.org)

Implementation: Machine learning analysis and multiple quadratic regression

Imène Garali^1,2*^ and Mourad Sahbatou^3*^

# I. Machine learning analysis with R

## I.1. Loading R packages

#Packages
library(knitr)
library(e1071)
library(Metrics)
library(gbm)
library("factoextra")
library(ggplot2)

## I.2. Importing datasets

We used publicly available DNA methylation data of seven CpGs located in the *ELOVL2* promoter from 1413 individuals aged between 0 to 91 years taken from four independent previously published studies. The data were divided into a training set (1028 individuals) and a testing set (385 individuals).

### I.2.1 Importing training datasets

dataTrain=read.table(".//Supplementary_Dataset_2.txt")
dataTrain=dataTrain[-1,-1]
colnames(dataTrain)=c("Age", "CpG_01", "CpG_02", "CpG_03", "CpG_04", "CpG_05", "CpG_06", "CpG_07")
dataTrain= apply(dataTrain, 2, function(x) as.numeric(as.character(x)))

print(kable(head(dataTrain)))

| Age | CpG_01 | CpG_02 | CpG_03 | CpG_04 | CpG_05 | CpG_06 | CpG_07 |
| --- | --- | --- | --- | --- | --- | --- | --- |
| 11 | 17.42 | 17.52 | 37.46 | 22.54 | 13.12 | 30.33 | 36.41 |
| 11 | 20.42 | 20.84 | 46.36 | 28.55 | 19.61 | 38.66 | 58.17 |
| 11 | 19.20 | 19.93 | 45.50 | 22.61 | 17.25 | 37.04 | 47.02 |
| 11 | 20.76 | 19.62 | 43.73 | 26.12 | 16.43 | 34.55 | 43.90 |
| 11 | 23.54 | 22.65 | 48.48 | 31.11 | 19.23 | 39.03 | 50.74 |
| 11 | 19.68 | 17.91 | 42.39 | 22.21 | 15.94 | 32.43 | 38.81 |

### I.2.2 Importing testing datasets

dataTest=read.table(".//Supplementary_Dataset_4.txt")
dataTest=dataTest[-1,-1]
colnames(dataTest)=c("Age", "CpG_01", "CpG_02", "CpG_03", "CpG_04", "CpG_05", "CpG_06", "CpG_07")
dataTest= apply(dataTest, 2, function(x) as.numeric(as.character(x)))

print(kable(head(dataTest)))

| Age | CpG_01 | CpG_02 | CpG_03 | CpG_04 | CpG_05 | CpG_06 | CpG_07 |
| --- | --- | --- | --- | --- | --- | --- | --- |
| 11 | 18.07 | 19.87 | 42.61 | 25.67 | 15.82 | 33.34 | 40.44 |
| 11 | 20.22 | 19.63 | 43.86 | 21.70 | 18.18 | 35.22 | 46.03 |
| 11 | 21.59 | 20.28 | 44.49 | 25.46 | 17.07 | 35.44 | 47.54 |
| 11 | 41.29 | 39.12 | 71.88 | 46.09 | 39.48 | 66.22 | 75.96 |
| 12 | 17.53 | 16.85 | 40.11 | 23.81 | 14.31 | 33.02 | 41.66 |
| 12 | 19.26 | 20.36 | 44.72 | 27.79 | 18.47 | 36.06 | 42.11 |

## I.3 Analysis.

For each age prediction from all possible combinations of the 7 CpGs (127 in total), the prediction accuracy was evaluated using the mean absolute deviation (MAD) and root mean square error (RMSE), while the correlation analyses were assessed using the Pearson R correlation coefficient.

### I.3.1 Support Vector Machine with radial (SVMr) function.

You can change analysis, depending on the kernel type.

dataTestSVM=dataTest
tabSVM=matrix(data = 0, nrow = 4, ncol = 127)
tabSVM=as.data.frame(tabSVM)
rownames(tabSVM)=c("MAE", "MeAE", "RMSE", "Cor")

dataTrainSVM=dataTrain
tabSVMT=matrix(data = 0, nrow = 4, ncol = 127)
tabSVMT=as.data.frame(tabSVMT)
rownames(tabSVMT)=c("MAE", "MeAE", "RMSE", "Cor")

t=1
for(i in 1:7)
{
 for(j in 1:dim(combn(2:8, i))[2])
 {
 nameCol="age"
 ypred=0
 for(k in 1:length(combn(1:7, i)[,j]))
 nameCol=paste(nameCol,combn(1:7, i)[,j][k], sep="-")

 # kernel="radial" or kernel="linear" or kernel="polynomial"
 regressor <- svm(formula = V1 ~., data = as.data.frame(cbind(dataTrain[,1],dataTrain[,combn(2:8, i)[,j]])),kernel="radial")
 dataPredect=as.matrix(dataTest[,combn(2:8, i)[,j]])
 dataPredectTrain=as.matrix(dataTrain[,combn(2:8, i)[,j]])

 if(i==1)
 {
 colnames(dataPredect)=c("V2")
 colnames(dataPredectTrain)=c("V2")
 }

 ypred = predict(regressor,dataPredect)
 dataTestSVM=cbind(as.matrix(dataTestSVM),ypred)
 error=ypred-as.numeric(as.character(dataTest[,1]))

 ypredTrain = predict(regressor,dataPredectTrain)
 dataTrainSVM=cbind(as.matrix(dataTrainSVM),ypredTrain)
 errorT=ypredTrain-as.numeric(as.character(dataTrain[,1]))


 colnames(dataTestSVM)[dim(dataTestSVM)[2]]=nameCol
 colnames(dataTrainSVM)[dim(dataTrainSVM)[2]]=nameCol

 tabSVM[1,t]= mean(abs(error))
 tabSVM[2,t]=median(abs(error))
 tabSVM[3,t]=rmse(as.numeric(as.character(dataTest[,1])), ypred)
 tabSVM[4,t]=cor(as.numeric(as.character(dataTest[,1])), ypred)
 colnames(tabSVM)[t]=nameCol

 tabSVMT[1,t]= mean(abs(errorT))
 tabSVMT[2,t]=median(abs(errorT))
 tabSVMT[3,t]=rmse(as.numeric(as.character(dataTrain[,1])), ypredTrain)
 tabSVMT[4,t]=cor(as.numeric(as.character(dataTrain[,1])), ypredTrain)
 colnames(tabSVMT)[t]=nameCol

 t=t+1

 }
}

#### i) Age prediction with training set

print(kable(t(tabSVM)))

|  | MAE | MeAE | RMSE | Cor |
| --- | --- | --- | --- | --- |
| age-1 | 6.332012 | 4.584324 | 8.926577 | 0.9105956 |
| age-2 | 6.720908 | 4.844894 | 9.285381 | 0.9027184 |
| age-3 | 7.669655 | 5.240251 | 10.619593 | 0.8737302 |
| age-4 | 7.837061 | 5.852521 | 10.401055 | 0.8774295 |
| age-5 | 6.049728 | 4.347377 | 8.323528 | 0.9233598 |
| age-6 | 4.845908 | 3.305793 | 7.180003 | 0.9427384 |
| age-7 | 6.055241 | 4.192418 | 8.587143 | 0.9174378 |
| age-1-2 | 6.083682 | 4.386034 | 8.514920 | 0.9190621 |
| age-1-3 | 5.943591 | 4.072180 | 8.445806 | 0.9205961 |
| age-1-4 | 5.904907 | 4.095870 | 8.363230 | 0.9221804 |
| age-1-5 | 5.396488 | 4.019895 | 7.604351 | 0.9363010 |
| age-1-6 | 4.851065 | 3.307549 | 7.008903 | 0.9454161 |
| age-1-7 | 5.356916 | 3.651244 | 7.539209 | 0.9369077 |
| age-2-3 | 6.265999 | 4.187596 | 8.901599 | 0.9113387 |
| age-2-4 | 5.999103 | 4.048256 | 8.541206 | 0.9181749 |
| age-2-5 | 5.278314 | 3.941829 | 7.477920 | 0.9379113 |
| age-2-6 | 4.714069 | 3.441130 | 6.817593 | 0.9485047 |
| age-2-7 | 5.152943 | 3.591576 | 7.418221 | 0.9387988 |
| age-3-4 | 7.046536 | 4.723482 | 9.680066 | 0.8955795 |
| age-3-5 | 5.364224 | 3.888783 | 7.603934 | 0.9362642 |
| age-3-6 | 4.738523 | 3.213278 | 6.928447 | 0.9466959 |
| age-3-7 | 5.943103 | 4.082498 | 8.418608 | 0.9209737 |
| age-4-5 | 5.371646 | 3.875995 | 7.557945 | 0.9368006 |
| age-4-6 | 4.786861 | 3.296592 | 6.920878 | 0.9468401 |
| age-4-7 | 6.034467 | 4.140843 | 8.453962 | 0.9198854 |
| age-5-6 | 4.552011 | 3.157531 | 6.638601 | 0.9512199 |
| age-5-7 | 5.017831 | 3.375413 | 7.224376 | 0.9424304 |
| age-6-7 | 4.700581 | 3.201332 | 6.832687 | 0.9482135 |
| age-1-2-3 | 5.792524 | 3.959520 | 8.228079 | 0.9245333 |
| age-1-2-4 | 5.749472 | 3.987624 | 8.173146 | 0.9254076 |
| age-1-2-5 | 5.250923 | 3.789272 | 7.489217 | 0.9380786 |
| age-1-2-6 | 4.782972 | 3.203333 | 6.906987 | 0.9470309 |
| age-1-2-7 | 5.177136 | 3.523843 | 7.445655 | 0.9383937 |
| age-1-3-4 | 5.827938 | 3.950683 | 8.292810 | 0.9234400 |
| age-1-3-5 | 5.172471 | 3.653002 | 7.399724 | 0.9397881 |
| age-1-3-6 | 4.787683 | 3.324623 | 6.980462 | 0.9458840 |
| age-1-3-7 | 5.313306 | 3.609457 | 7.552847 | 0.9368807 |
| age-1-4-5 | 5.145322 | 3.648642 | 7.392901 | 0.9397932 |
| age-1-4-6 | 4.880030 | 3.539938 | 7.050726 | 0.9447885 |
| age-1-4-7 | 5.327764 | 3.580694 | 7.545531 | 0.9366928 |
| age-1-5-6 | 4.660086 | 3.133355 | 6.743456 | 0.9496507 |
| age-1-5-7 | 4.916123 | 3.405135 | 7.027209 | 0.9456789 |
| age-1-6-7 | 4.760363 | 3.099419 | 6.892289 | 0.9472895 |
| age-2-3-4 | 5.967871 | 4.257817 | 8.524339 | 0.9185298 |
| age-2-3-5 | 5.045848 | 3.666699 | 7.283185 | 0.9411614 |
| age-2-3-6 | 4.751201 | 3.447209 | 6.940359 | 0.9465565 |
| age-2-3-7 | 5.116672 | 3.519361 | 7.434822 | 0.9384818 |
| age-2-4-5 | 5.034712 | 3.628788 | 7.212765 | 0.9422582 |
| age-2-4-6 | 4.677718 | 3.323462 | 6.835576 | 0.9481698 |
| age-2-4-7 | 5.130105 | 3.692821 | 7.494576 | 0.9373780 |
| age-2-5-6 | 4.534375 | 3.259354 | 6.613541 | 0.9515777 |
| age-2-5-7 | 4.749229 | 3.182441 | 6.982092 | 0.9461723 |
| age-2-6-7 | 4.552887 | 3.085383 | 6.674106 | 0.9506471 |
| age-3-4-5 | 5.239445 | 3.882343 | 7.507711 | 0.9373815 |
| age-3-4-6 | 4.789163 | 3.211789 | 6.963125 | 0.9461431 |
| age-3-4-7 | 5.934358 | 3.961661 | 8.454615 | 0.9202272 |
| age-3-5-6 | 4.500459 | 3.088772 | 6.612856 | 0.9516061 |
| age-3-5-7 | 4.954359 | 3.344552 | 7.229517 | 0.9422292 |
| age-3-6-7 | 4.656625 | 3.048980 | 6.833628 | 0.9481868 |
| age-4-5-6 | 4.529406 | 3.332591 | 6.607393 | 0.9517023 |
| age-4-5-7 | 5.024603 | 3.290879 | 7.191515 | 0.9428715 |
| age-4-6-7 | 4.722759 | 3.314560 | 6.868446 | 0.9476429 |
| age-5-6-7 | 4.510079 | 3.152845 | 6.551955 | 0.9525044 |
| age-1-2-3-4 | 5.676691 | 3.959604 | 8.132412 | 0.9262219 |
| age-1-2-3-5 | 5.068511 | 3.649504 | 7.337761 | 0.9404634 |
| age-1-2-3-6 | 4.746129 | 3.338485 | 6.917410 | 0.9468764 |
| age-1-2-3-7 | 5.145910 | 3.318887 | 7.455916 | 0.9381913 |
| age-1-2-4-5 | 5.067212 | 3.531243 | 7.348999 | 0.9402923 |
| age-1-2-4-6 | 4.759232 | 3.304895 | 6.919949 | 0.9468557 |
| age-1-2-4-7 | 5.176392 | 3.646402 | 7.459810 | 0.9381219 |
| age-1-2-5-6 | 4.594121 | 3.354545 | 6.632060 | 0.9513188 |
| age-1-2-5-7 | 4.781558 | 3.268320 | 7.010447 | 0.9457490 |
| age-1-2-6-7 | 4.667614 | 3.079740 | 6.812311 | 0.9485155 |
| age-1-3-4-5 | 5.127258 | 3.684414 | 7.348287 | 0.9405289 |
| age-1-3-4-6 | 4.826123 | 3.325180 | 7.034769 | 0.9450283 |
| age-1-3-4-7 | 5.342115 | 3.679757 | 7.621261 | 0.9354962 |
| age-1-3-5-6 | 4.580422 | 3.190864 | 6.688950 | 0.9504631 |
| age-1-3-5-7 | 4.827661 | 3.328298 | 6.932286 | 0.9472793 |
| age-1-3-6-7 | 4.730685 | 3.263110 | 6.894641 | 0.9472635 |
| age-1-4-5-6 | 4.664674 | 3.375839 | 6.713879 | 0.9501078 |
| age-1-4-5-7 | 4.881335 | 3.484120 | 7.013541 | 0.9456729 |
| age-1-4-6-7 | 4.803424 | 3.176786 | 6.946152 | 0.9464509 |
| age-1-5-6-7 | 4.600417 | 3.179463 | 6.673678 | 0.9506705 |
| age-2-3-4-5 | 4.938500 | 3.571029 | 7.176550 | 0.9428580 |
| age-2-3-4-6 | 4.707597 | 3.284711 | 6.911477 | 0.9469949 |
| age-2-3-4-7 | 5.117840 | 3.600558 | 7.505029 | 0.9372529 |
| age-2-3-5-6 | 4.487588 | 3.159136 | 6.602012 | 0.9517340 |
| age-2-3-5-7 | 4.640074 | 3.315602 | 6.859815 | 0.9480115 |
| age-2-3-6-7 | 4.612709 | 3.375265 | 6.756721 | 0.9494022 |
| age-2-4-5-6 | 4.464502 | 3.182224 | 6.538613 | 0.9526924 |
| age-2-4-5-7 | 4.674401 | 3.220464 | 6.977931 | 0.9461081 |
| age-2-4-6-7 | 4.593067 | 3.085704 | 6.792587 | 0.9488336 |
| age-2-5-6-7 | 4.467575 | 3.140058 | 6.560924 | 0.9523746 |
| age-3-4-5-6 | 4.547287 | 3.070064 | 6.672379 | 0.9506889 |
| age-3-4-5-7 | 4.970266 | 3.288251 | 7.238368 | 0.9420057 |
| age-3-4-6-7 | 4.714489 | 3.155152 | 6.924635 | 0.9467549 |
| age-3-5-6-7 | 4.452712 | 3.030232 | 6.546381 | 0.9526197 |
| age-4-5-6-7 | 4.494712 | 3.198557 | 6.558546 | 0.9524114 |
| age-1-2-3-4-5 | 4.971072 | 3.438634 | 7.251140 | 0.9418015 |
| age-1-2-3-4-6 | 4.766204 | 3.230484 | 6.927401 | 0.9467173 |
| age-1-2-3-4-7 | 5.165134 | 3.497361 | 7.490057 | 0.9375656 |
| age-1-2-3-5-6 | 4.557405 | 3.277960 | 6.611940 | 0.9515885 |
| age-1-2-3-5-7 | 4.680047 | 3.226919 | 6.899381 | 0.9475016 |
| age-1-2-3-6-7 | 4.646347 | 3.125590 | 6.793201 | 0.9488192 |
| age-1-2-4-5-6 | 4.611512 | 3.357860 | 6.634507 | 0.9512963 |
| age-1-2-4-5-7 | 4.755018 | 3.112420 | 6.994252 | 0.9458961 |
| age-1-2-4-6-7 | 4.666504 | 3.008410 | 6.843814 | 0.9480423 |
| age-1-2-5-6-7 | 4.516134 | 3.262951 | 6.619726 | 0.9515181 |
| age-1-3-4-5-6 | 4.623351 | 3.348094 | 6.705815 | 0.9502053 |
| age-1-3-4-5-7 | 4.852465 | 3.349132 | 7.000948 | 0.9460758 |
| age-1-3-4-6-7 | 4.767558 | 3.133177 | 6.959131 | 0.9462466 |
| age-1-3-5-6-7 | 4.521154 | 3.029287 | 6.588247 | 0.9519578 |
| age-1-4-5-6-7 | 4.609297 | 3.285522 | 6.673825 | 0.9506808 |
| age-2-3-4-5-6 | 4.465423 | 3.029274 | 6.580100 | 0.9520709 |
| age-2-3-4-5-7 | 4.658159 | 3.156693 | 6.939498 | 0.9467219 |
| age-2-3-4-6-7 | 4.611853 | 3.179730 | 6.817032 | 0.9484552 |
| age-2-3-5-6-7 | 4.410068 | 3.010881 | 6.491947 | 0.9533819 |
| age-2-4-5-6-7 | 4.454544 | 3.019431 | 6.578880 | 0.9520955 |
| age-3-4-5-6-7 | 4.492959 | 3.114956 | 6.593189 | 0.9519059 |
| age-1-2-3-4-5-6 | 4.576646 | 3.271902 | 6.639668 | 0.9511857 |
| age-1-2-3-4-5-7 | 4.694282 | 2.974202 | 6.928897 | 0.9469108 |
| age-1-2-3-4-6-7 | 4.685758 | 3.017835 | 6.863708 | 0.9477269 |
| age-1-2-3-5-6-7 | 4.463579 | 3.243755 | 6.543529 | 0.9526233 |
| age-1-2-4-5-6-7 | 4.555044 | 3.170688 | 6.652206 | 0.9509988 |
| age-1-3-4-5-6-7 | 4.574135 | 3.127841 | 6.644088 | 0.9511396 |
| age-2-3-4-5-6-7 | 4.411344 | 3.033743 | 6.524636 | 0.9528784 |
| age-1-2-3-4-5-6-7 | 4.515609 | 3.193331 | 6.616109 | 0.9515176 |

### ii) Age prediction with testing set

print(kable(t(tabSVMT)))

|  | MAE | MeAE | RMSE | Cor |
| --- | --- | --- | --- | --- |
| age-1 | 6.138145 | 4.751348 | 8.204849 | 0.9224956 |
| age-2 | 6.880484 | 5.251264 | 9.294331 | 0.9004789 |
| age-3 | 7.919116 | 5.814790 | 10.719515 | 0.8661155 |
| age-4 | 8.377949 | 6.488694 | 10.940401 | 0.8585221 |
| age-5 | 6.754189 | 5.254207 | 9.143981 | 0.9035170 |
| age-6 | 5.315243 | 3.773549 | 7.604496 | 0.9340283 |
| age-7 | 6.898284 | 5.001454 | 9.273950 | 0.9001925 |
| age-1-2 | 5.851190 | 4.362806 | 7.853015 | 0.9292822 |
| age-1-3 | 5.749270 | 4.101053 | 7.723990 | 0.9316447 |
| age-1-4 | 5.710078 | 4.241097 | 7.692249 | 0.9321550 |
| age-1-5 | 5.510214 | 4.237046 | 7.264449 | 0.9397528 |
| age-1-6 | 4.918861 | 3.647676 | 6.677774 | 0.9493160 |
| age-1-7 | 5.449986 | 3.974969 | 7.277386 | 0.9395136 |
| age-2-3 | 6.324535 | 4.491313 | 8.728580 | 0.9125939 |
| age-2-4 | 6.268080 | 4.579180 | 8.609659 | 0.9147925 |
| age-2-5 | 5.596082 | 4.260348 | 7.500650 | 0.9356121 |
| age-2-6 | 4.980689 | 3.533016 | 6.805174 | 0.9473318 |
| age-2-7 | 5.659155 | 4.119016 | 7.620975 | 0.9334715 |
| age-3-4 | 7.314333 | 5.493430 | 9.827473 | 0.8878528 |
| age-3-5 | 5.981381 | 4.314218 | 8.050307 | 0.9256915 |
| age-3-6 | 5.101082 | 3.560018 | 7.172485 | 0.9413841 |
| age-3-7 | 6.404927 | 4.694473 | 8.646594 | 0.9136156 |
| age-4-5 | 5.832547 | 4.363348 | 7.871772 | 0.9288593 |
| age-4-6 | 5.080722 | 3.677195 | 6.915857 | 0.9455433 |
| age-4-7 | 6.733691 | 4.969054 | 8.990512 | 0.9061023 |
| age-5-6 | 4.970681 | 3.516542 | 6.838491 | 0.9467986 |
| age-5-7 | 5.589879 | 3.962322 | 7.605585 | 0.9338583 |
| age-6-7 | 5.051489 | 3.713544 | 6.843451 | 0.9467132 |
| age-1-2-3 | 5.567313 | 3.920181 | 7.498344 | 0.9356927 |
| age-1-2-4 | 5.562613 | 4.060662 | 7.515733 | 0.9353417 |
| age-1-2-5 | 5.313266 | 4.100356 | 7.068137 | 0.9430717 |
| age-1-2-6 | 4.857880 | 3.537449 | 6.627681 | 0.9500937 |
| age-1-2-7 | 5.254806 | 3.871375 | 7.044725 | 0.9434222 |
| age-1-3-4 | 5.607862 | 4.075350 | 7.571517 | 0.9343748 |
| age-1-3-5 | 5.282067 | 3.939280 | 7.094838 | 0.9426502 |
| age-1-3-6 | 4.856654 | 3.455822 | 6.670368 | 0.9494399 |
| age-1-3-7 | 5.294418 | 3.777999 | 7.140717 | 0.9418497 |
| age-1-4-5 | 5.258099 | 3.897999 | 7.039769 | 0.9435204 |
| age-1-4-6 | 4.848365 | 3.512949 | 6.617876 | 0.9502450 |
| age-1-4-7 | 5.310036 | 3.792624 | 7.142659 | 0.9417999 |
| age-1-5-6 | 4.821208 | 3.511496 | 6.522705 | 0.9517126 |
| age-1-5-7 | 5.141883 | 3.881441 | 6.836706 | 0.9468256 |
| age-1-6-7 | 4.826882 | 3.546264 | 6.571964 | 0.9509521 |
| age-2-3-4 | 6.167978 | 4.455209 | 8.521615 | 0.9165696 |
| age-2-3-5 | 5.351247 | 3.796681 | 7.304692 | 0.9390917 |
| age-2-3-6 | 4.879492 | 3.521985 | 6.723723 | 0.9486058 |
| age-2-3-7 | 5.516753 | 3.974141 | 7.481525 | 0.9359501 |
| age-2-4-5 | 5.292884 | 3.978605 | 7.237286 | 0.9402034 |
| age-2-4-6 | 4.920163 | 3.595154 | 6.725503 | 0.9485750 |
| age-2-4-7 | 5.532478 | 3.907452 | 7.498594 | 0.9356596 |
| age-2-5-6 | 4.801458 | 3.439210 | 6.602956 | 0.9504754 |
| age-2-5-7 | 5.135964 | 3.769504 | 6.932442 | 0.9453057 |
| age-2-6-7 | 4.851237 | 3.490623 | 6.627848 | 0.9501002 |
| age-3-4-5 | 5.584077 | 4.089956 | 7.554476 | 0.9346977 |
| age-3-4-6 | 4.989754 | 3.623548 | 6.880845 | 0.9461410 |
| age-3-4-7 | 6.312494 | 4.586337 | 8.563036 | 0.9153870 |
| age-3-5-6 | 4.888333 | 3.395992 | 6.785658 | 0.9476405 |
| age-3-5-7 | 5.364427 | 3.796993 | 7.352868 | 0.9382345 |
| age-3-6-7 | 4.920514 | 3.533838 | 6.762383 | 0.9480119 |
| age-4-5-6 | 4.872588 | 3.483470 | 6.632886 | 0.9500251 |
| age-4-5-7 | 5.475046 | 4.014269 | 7.468224 | 0.9362265 |
| age-4-6-7 | 5.008844 | 3.761223 | 6.810691 | 0.9472240 |
| age-5-6-7 | 4.809453 | 3.389650 | 6.525801 | 0.9516553 |
| age-1-2-3-4 | 5.467899 | 3.985314 | 7.426378 | 0.9369516 |
| age-1-2-3-5 | 5.086335 | 3.726750 | 6.914983 | 0.9455675 |
| age-1-2-3-6 | 4.761202 | 3.289352 | 6.540937 | 0.9514240 |
| age-1-2-3-7 | 5.131241 | 3.728522 | 6.921893 | 0.9454332 |
| age-1-2-4-5 | 5.091924 | 3.717266 | 6.899582 | 0.9458151 |
| age-1-2-4-6 | 4.798763 | 3.363883 | 6.563077 | 0.9511024 |
| age-1-2-4-7 | 5.155608 | 3.729630 | 6.958114 | 0.9448478 |
| age-1-2-5-6 | 4.735401 | 3.424430 | 6.448550 | 0.9528387 |
| age-1-2-5-7 | 4.953089 | 3.707300 | 6.657538 | 0.9496477 |
| age-1-2-6-7 | 4.758289 | 3.484581 | 6.479441 | 0.9523566 |
| age-1-3-4-5 | 5.137622 | 3.835768 | 6.938692 | 0.9452427 |
| age-1-3-4-6 | 4.785385 | 3.398857 | 6.554790 | 0.9512146 |
| age-1-3-4-7 | 5.234016 | 3.832807 | 7.072901 | 0.9429669 |
| age-1-3-5-6 | 4.736757 | 3.360053 | 6.485717 | 0.9522681 |
| age-1-3-5-7 | 4.942661 | 3.480901 | 6.686531 | 0.9492582 |
| age-1-3-6-7 | 4.742472 | 3.393870 | 6.513168 | 0.9518528 |
| age-1-4-5-6 | 4.743951 | 3.346865 | 6.455882 | 0.9527201 |
| age-1-4-5-7 | 4.977520 | 3.605474 | 6.728957 | 0.9485168 |
| age-1-4-6-7 | 4.797542 | 3.484380 | 6.537031 | 0.9514835 |
| age-1-5-6-7 | 4.684387 | 3.436226 | 6.359224 | 0.9541640 |
| age-2-3-4-5 | 5.202335 | 3.835674 | 7.145033 | 0.9417753 |
| age-2-3-4-6 | 4.853701 | 3.510167 | 6.664615 | 0.9495201 |
| age-2-3-4-7 | 5.465635 | 3.844093 | 7.422975 | 0.9369941 |
| age-2-3-5-6 | 4.733545 | 3.366767 | 6.533397 | 0.9515470 |
| age-2-3-5-7 | 4.929889 | 3.521844 | 6.766519 | 0.9479647 |
| age-2-3-6-7 | 4.741907 | 3.425953 | 6.516089 | 0.9518011 |
| age-2-4-5-6 | 4.748107 | 3.436994 | 6.525799 | 0.9516548 |
| age-2-4-5-7 | 4.950071 | 3.537936 | 6.787951 | 0.9476366 |
| age-2-4-6-7 | 4.819507 | 3.427892 | 6.574213 | 0.9509182 |
| age-2-5-6-7 | 4.662988 | 3.426287 | 6.374890 | 0.9539198 |
| age-3-4-5-6 | 4.811437 | 3.418333 | 6.603380 | 0.9504735 |
| age-3-4-5-7 | 5.294741 | 3.852636 | 7.285056 | 0.9394084 |
| age-3-4-6-7 | 4.887737 | 3.513930 | 6.703465 | 0.9489267 |
| age-3-5-6-7 | 4.726896 | 3.410729 | 6.469502 | 0.9525084 |
| age-4-5-6-7 | 4.765172 | 3.445225 | 6.477472 | 0.9524063 |
| age-1-2-3-4-5 | 4.998079 | 3.569520 | 6.817095 | 0.9471449 |
| age-1-2-3-4-6 | 4.750601 | 3.338389 | 6.510579 | 0.9518896 |
| age-1-2-3-4-7 | 5.098556 | 3.578334 | 6.903560 | 0.9457321 |
| age-1-2-3-5-6 | 4.648640 | 3.277286 | 6.379510 | 0.9538509 |
| age-1-2-3-5-7 | 4.787794 | 3.406770 | 6.520312 | 0.9517969 |
| age-1-2-3-6-7 | 4.678285 | 3.205934 | 6.420490 | 0.9532567 |
| age-1-2-4-5-6 | 4.687309 | 3.357433 | 6.398995 | 0.9535799 |
| age-1-2-4-5-7 | 4.836712 | 3.466573 | 6.571333 | 0.9509892 |
| age-1-2-4-6-7 | 4.729087 | 3.358370 | 6.458507 | 0.9526842 |
| age-1-2-5-6-7 | 4.620598 | 3.366797 | 6.269106 | 0.9554812 |
| age-1-3-4-5-6 | 4.694365 | 3.335814 | 6.417096 | 0.9532923 |
| age-1-3-4-5-7 | 4.888959 | 3.529193 | 6.653858 | 0.9497725 |
| age-1-3-4-6-7 | 4.728194 | 3.420435 | 6.469039 | 0.9525232 |
| age-1-3-5-6-7 | 4.614275 | 3.288539 | 6.316604 | 0.9547798 |
| age-1-4-5-6-7 | 4.646276 | 3.323075 | 6.339903 | 0.9544348 |
| age-2-3-4-5-6 | 4.706787 | 3.354035 | 6.473455 | 0.9524464 |
| age-2-3-4-5-7 | 4.877353 | 3.454301 | 6.722591 | 0.9487222 |
| age-2-3-4-6-7 | 4.741205 | 3.200057 | 6.499933 | 0.9520513 |
| age-2-3-5-6-7 | 4.613891 | 3.213664 | 6.325704 | 0.9546426 |
| age-2-4-5-6-7 | 4.623565 | 3.265121 | 6.346164 | 0.9543711 |
| age-3-4-5-6-7 | 4.709874 | 3.354945 | 6.441784 | 0.9529385 |
| age-1-2-3-4-5-6 | 4.638506 | 3.278814 | 6.354157 | 0.9542316 |
| age-1-2-3-4-5-7 | 4.770533 | 3.436367 | 6.517925 | 0.9518246 |
| age-1-2-3-4-6-7 | 4.670225 | 3.237150 | 6.413539 | 0.9533599 |
| age-1-2-3-5-6-7 | 4.554645 | 3.185487 | 6.229147 | 0.9560519 |
| age-1-2-4-5-6-7 | 4.587746 | 3.244048 | 6.251037 | 0.9557410 |
| age-1-3-4-5-6-7 | 4.603253 | 3.266509 | 6.308292 | 0.9549031 |
| age-2-3-4-5-6-7 | 4.603163 | 3.229119 | 6.311785 | 0.9548480 |
| age-1-2-3-4-5-6-7 | 4.558136 | 3.163116 | 6.236446 | 0.9559449 |

### I.3.2 Gradient Boosting Regressor (GBR)

dataTestgbm=dataTest
dataTraingbm=dataTrain

tabGBM=matrix(data = 0, nrow = 4, ncol = 127)
tabGBM=as.data.frame(tabGBM)
rownames(tabGBM)=c("MAE", "MeAE", "RMSE", "Cor")

tabGBMT=matrix(data = 0, nrow = 4, ncol = 127)
tabGBMT=as.data.frame(tabGBMT)
rownames(tabGBMT)=c("MAE", "MeAE", "RMSE", "Cor")

error1 = error1T =0
error2 = error2T =0
error3 = error3T =0
error4 = error4T =0

t=1
for(i in 1:7)
{
 for(j in 1:dim(combn(2:8, i))[2])
 {

 nameCol="age"
 ypred=0
 for(k in 1:length(combn(1:7, i)[,j]))
 nameCol=paste(nameCol,combn(1:7, i)[,j][k], sep="-")

 regressor=gbm(V1 ~. ,data = as.data.frame(cbind(dataTrain[,1],dataTrain[,combn(2:8, i)[,j]])),distribution = "gaussian",n.trees = 10000,
 shrinkage = 0.01, interaction.depth = 4)
 dataPredect=as.matrix(dataTest[,combn(2:8, i)[,j]])
 dataPredectTrain=as.matrix(dataTrain[,combn(2:8, i)[,j]])

 if(i==1)
 {
 colnames(dataPredect)=c("V2")
 colnames(dataPredectTrain)=c("V2")
 }

 n.trees = seq(from=100 ,to=10000, by=100)

 #Generating a Prediction matrix for each Tree
 predmatrix<-predict(regressor,as.data.frame(dataPredect),n.trees = n.trees)
 predmatrixTrain<-predict(regressor,as.data.frame(dataPredectTrain),n.trees = n.trees)

 #Calculating The Mean squared Test Error
 error1<-with(as.data.frame(dataTest),apply( abs(predmatrix-as.numeric(as.character(dataTest[,1]))),2,mean))
 error2<-with(as.data.frame(dataTest),apply( abs(predmatrix-as.numeric(as.character(dataTest[,1]))),2,median))
 z=1
 for (z in 1:dim(predmatrix)[2])
 error3[z]<-rmse(as.numeric(as.character(dataTest[,1])), predmatrix[,z])
 z=1
 for (z in 1:dim(predmatrix)[2])
 error4[z]<-cor(as.numeric(as.character(dataTest[,1])), predmatrix[,z])

 error1T<-with(as.data.frame(dataTrain),apply( abs(predmatrixTrain-as.numeric(as.character(dataTrain[,1]))),2,mean))
 error2T<-with(as.data.frame(dataTrain),apply( abs(predmatrixTrain-as.numeric(as.character(dataTrain[,1]))),2,median))
 z=1
 for (z in 1:dim(predmatrixTrain)[2])
 error3T[z]<-rmse(as.numeric(as.character(dataTrain[,1])), predmatrixTrain[,z])
 z=1
 for (z in 1:dim(predmatrixTrain)[2])
 error4T[z]<-cor(as.numeric(as.character(dataTrain[,1])), predmatrixTrain[,z])

 dataTestgbm=cbind(dataTestgbm,predmatrix[,names(which(error1== min(error1),arr.ind=TRUE))])
 dataTraingbm=cbind(dataTraingbm,predmatrixTrain[,names(which(error1T== min(error1T),arr.ind=TRUE))])
 colnames(dataTestgbm)[dim(dataTestgbm)[2]]=nameCol
 colnames(dataTraingbm)[dim(dataTraingbm)[2]]=nameCol


 colnames(dataTestgbm)[dim(dataTestgbm)[2]]=nameCol
 tabGBM[1,t]= min(error1)
 tabGBM[2,t]=min(error2)
 tabGBM[3,t]=min(error3)
 tabGBM[4,t]=max(error4)
 colnames(tabGBM)[t]=nameCol

 tabGBMT[1,t]= min(error1T)
 tabGBMT[2,t]=min(error2T)
 tabGBMT[3,t]=min(error3T)
 tabGBMT[4,t]=max(error4T)
 colnames(tabGBMT)[t]=nameCol
 t=t+1

 }
}

#### i) Age prediction with training set

print(kable(t(tabGBMT)))

|  | MAE | MeAE | RMSE | Cor |
| --- | --- | --- | --- | --- |
| age-1 | 4.964214 | 3.659535 | 6.871025 | 0.9463205 |
| age-2 | 5.824430 | 4.417964 | 7.945896 | 0.9275177 |
| age-3 | 6.276899 | 4.648068 | 8.504440 | 0.9165968 |
| age-4 | 6.548406 | 5.077608 | 8.509860 | 0.9165808 |
| age-5 | 5.499477 | 4.262378 | 7.452276 | 0.9365759 |
| age-6 | 4.492943 | 3.287317 | 6.409397 | 0.9534770 |
| age-7 | 5.463333 | 4.214269 | 7.161123 | 0.9416570 |
| age-1-2 | 4.286413 | 3.074528 | 5.912108 | 0.9606232 |
| age-1-3 | 4.063391 | 3.010945 | 5.457402 | 0.9665984 |
| age-1-4 | 4.086536 | 3.193220 | 5.509348 | 0.9659352 |
| age-1-5 | 3.756962 | 2.849809 | 5.087910 | 0.9710022 |
| age-1-6 | 3.465854 | 2.506991 | 4.658929 | 0.9757428 |
| age-1-7 | 3.756355 | 2.877388 | 4.890601 | 0.9732848 |
| age-2-3 | 4.618428 | 3.360592 | 6.403258 | 0.9536851 |
| age-2-4 | 4.575855 | 3.253959 | 6.292792 | 0.9553506 |
| age-2-5 | 3.929622 | 2.931466 | 5.332846 | 0.9681340 |
| age-2-6 | 3.611977 | 2.708247 | 4.857640 | 0.9736191 |
| age-2-7 | 4.078096 | 3.048697 | 5.395589 | 0.9673862 |
| age-3-4 | 4.920771 | 3.525438 | 6.654589 | 0.9500397 |
| age-3-5 | 4.024426 | 3.156448 | 5.363899 | 0.9677817 |
| age-3-6 | 3.566472 | 2.602745 | 4.941046 | 0.9726863 |
| age-3-7 | 4.204649 | 3.362738 | 5.511387 | 0.9660411 |
| age-4-5 | 3.969114 | 3.044965 | 5.282687 | 0.9687643 |
| age-4-6 | 3.592128 | 2.626121 | 4.812149 | 0.9741314 |
| age-4-7 | 4.367972 | 3.300128 | 5.706720 | 0.9635261 |
| age-5-6 | 3.509516 | 2.584408 | 4.711302 | 0.9752020 |
| age-5-7 | 3.810097 | 2.892672 | 5.095079 | 0.9709574 |
| age-6-7 | 3.483283 | 2.598486 | 4.679706 | 0.9755409 |
| age-1-2-3 | 3.558440 | 2.646716 | 4.850127 | 0.9737754 |
| age-1-2-4 | 3.560194 | 2.654811 | 4.845325 | 0.9738414 |
| age-1-2-5 | 3.278036 | 2.527239 | 4.461479 | 0.9778423 |
| age-1-2-6 | 3.083277 | 2.373878 | 4.163649 | 0.9806991 |
| age-1-2-7 | 3.245502 | 2.546573 | 4.308193 | 0.9793716 |
| age-1-3-4 | 3.539200 | 2.821734 | 4.720982 | 0.9751838 |
| age-1-3-5 | 3.243445 | 2.482231 | 4.272834 | 0.9797192 |
| age-1-3-6 | 2.994592 | 2.334569 | 3.980439 | 0.9824261 |
| age-1-3-7 | 3.185700 | 2.480326 | 4.138707 | 0.9810038 |
| age-1-4-5 | 3.236074 | 2.444579 | 4.306361 | 0.9793800 |
| age-1-4-6 | 3.077780 | 2.282632 | 4.075561 | 0.9815422 |
| age-1-4-7 | 3.230922 | 2.562334 | 4.209445 | 0.9803250 |
| age-1-5-6 | 3.005514 | 2.350442 | 3.972543 | 0.9824653 |
| age-1-5-7 | 3.070737 | 2.370471 | 4.039240 | 0.9818761 |
| age-1-6-7 | 2.998459 | 2.267110 | 3.932361 | 0.9828309 |
| age-2-3-4 | 3.955132 | 2.746760 | 5.406949 | 0.9673744 |
| age-2-3-5 | 3.407555 | 2.594714 | 4.566071 | 0.9767970 |
| age-2-3-6 | 3.089461 | 2.277251 | 4.184279 | 0.9805384 |
| age-2-3-7 | 3.326889 | 2.526558 | 4.390634 | 0.9786090 |
| age-2-4-5 | 3.326200 | 2.568948 | 4.463160 | 0.9778542 |
| age-2-4-6 | 3.124897 | 2.384200 | 4.149860 | 0.9808587 |
| age-2-4-7 | 3.414514 | 2.610103 | 4.488567 | 0.9776307 |
| age-2-5-6 | 3.092305 | 2.306263 | 4.123886 | 0.9810940 |
| age-2-5-7 | 3.129875 | 2.332941 | 4.170120 | 0.9806785 |
| age-2-6-7 | 3.060870 | 2.256191 | 4.047808 | 0.9818056 |
| age-3-4-5 | 3.398648 | 2.669956 | 4.501348 | 0.9774586 |
| age-3-4-6 | 3.139308 | 2.278820 | 4.247521 | 0.9799544 |
| age-3-4-7 | 3.612098 | 2.666458 | 4.739777 | 0.9750662 |
| age-3-5-6 | 3.099799 | 2.336879 | 4.144219 | 0.9809143 |
| age-3-5-7 | 3.164453 | 2.440382 | 4.184507 | 0.9805618 |
| age-3-6-7 | 2.996362 | 2.174002 | 4.022366 | 0.9820440 |
| age-4-5-6 | 3.037485 | 2.481360 | 4.018343 | 0.9820738 |
| age-4-5-7 | 3.198154 | 2.478420 | 4.226607 | 0.9801727 |
| age-4-6-7 | 3.072057 | 2.348995 | 4.083661 | 0.9814811 |
| age-5-6-7 | 2.962777 | 2.321938 | 3.923679 | 0.9828994 |
| age-1-2-3-4 | 3.147035 | 2.392241 | 4.241765 | 0.9800446 |
| age-1-2-3-5 | 2.867359 | 2.221504 | 3.831120 | 0.9837547 |
| age-1-2-3-6 | 2.719432 | 2.118104 | 3.612356 | 0.9855620 |
| age-1-2-3-7 | 2.769604 | 2.128333 | 3.630444 | 0.9854492 |
| age-1-2-4-5 | 2.887361 | 2.280078 | 3.857438 | 0.9835290 |
| age-1-2-4-6 | 2.772787 | 2.109098 | 3.686291 | 0.9849550 |
| age-1-2-4-7 | 2.855109 | 2.290968 | 3.737444 | 0.9845574 |
| age-1-2-5-6 | 2.695094 | 2.030858 | 3.588712 | 0.9857305 |
| age-1-2-5-7 | 2.665732 | 2.035356 | 3.548146 | 0.9860850 |
| age-1-2-6-7 | 2.700628 | 2.045900 | 3.581611 | 0.9857984 |
| age-1-3-4-5 | 2.863090 | 2.227516 | 3.772142 | 0.9842616 |
| age-1-3-4-6 | 2.714346 | 2.060929 | 3.613398 | 0.9855582 |
| age-1-3-4-7 | 2.889983 | 2.283710 | 3.733875 | 0.9845908 |
| age-1-3-5-6 | 2.657874 | 2.107282 | 3.511366 | 0.9863749 |
| age-1-3-5-7 | 2.635642 | 2.040929 | 3.460420 | 0.9867767 |
| age-1-3-6-7 | 2.619014 | 2.054165 | 3.458264 | 0.9867858 |
| age-1-4-5-6 | 2.707014 | 2.103552 | 3.548240 | 0.9860705 |
| age-1-4-5-7 | 2.691316 | 2.097215 | 3.533243 | 0.9862090 |
| age-1-4-6-7 | 2.694247 | 2.085104 | 3.520501 | 0.9862943 |
| age-1-5-6-7 | 2.580568 | 2.044101 | 3.397659 | 0.9872405 |
| age-2-3-4-5 | 2.985729 | 2.353963 | 3.946048 | 0.9827727 |
| age-2-3-4-6 | 2.787136 | 2.100324 | 3.741334 | 0.9844937 |
| age-2-3-4-7 | 2.960903 | 2.323253 | 3.898833 | 0.9832086 |
| age-2-3-5-6 | 2.743252 | 2.035600 | 3.653720 | 0.9852278 |
| age-2-3-5-7 | 2.721073 | 2.125259 | 3.553743 | 0.9860275 |
| age-2-3-6-7 | 2.632449 | 1.964766 | 3.496143 | 0.9864905 |
| age-2-4-5-6 | 2.747013 | 2.169068 | 3.618592 | 0.9855132 |
| age-2-4-5-7 | 2.711656 | 2.135590 | 3.580087 | 0.9858420 |
| age-2-4-6-7 | 2.721060 | 2.111355 | 3.580961 | 0.9858128 |
| age-2-5-6-7 | 2.612725 | 1.964336 | 3.452143 | 0.9868125 |
| age-3-4-5-6 | 2.786047 | 2.133650 | 3.661489 | 0.9851748 |
| age-3-4-5-7 | 2.791639 | 2.102663 | 3.694264 | 0.9848962 |
| age-3-4-6-7 | 2.749082 | 2.089477 | 3.656945 | 0.9852157 |
| age-3-5-6-7 | 2.631454 | 1.936130 | 3.502749 | 0.9864261 |
| age-4-5-6-7 | 2.662636 | 2.138799 | 3.481507 | 0.9866030 |
| age-1-2-3-4-5 | 2.587267 | 2.042491 | 3.411390 | 0.9871750 |
| age-1-2-3-4-6 | 2.507409 | 1.933332 | 3.304214 | 0.9879541 |
| age-1-2-3-4-7 | 2.557989 | 2.075210 | 3.327545 | 0.9878066 |
| age-1-2-3-5-6 | 2.424479 | 1.876796 | 3.227244 | 0.9885129 |
| age-1-2-3-5-7 | 2.357742 | 1.893277 | 3.111594 | 0.9893322 |
| age-1-2-3-6-7 | 2.351462 | 1.795816 | 3.116354 | 0.9893002 |
| age-1-2-4-5-6 | 2.470091 | 1.931925 | 3.244995 | 0.9883880 |
| age-1-2-4-5-7 | 2.402680 | 1.894036 | 3.152582 | 0.9890484 |
| age-1-2-4-6-7 | 2.475170 | 1.838065 | 3.243888 | 0.9884043 |
| age-1-2-5-6-7 | 2.318096 | 1.724521 | 3.085434 | 0.9895043 |
| age-1-3-4-5-6 | 2.440853 | 1.930393 | 3.225264 | 0.9885256 |
| age-1-3-4-5-7 | 2.417603 | 1.913641 | 3.163354 | 0.9889712 |
| age-1-3-4-6-7 | 2.432015 | 1.924266 | 3.182862 | 0.9888355 |
| age-1-3-5-6-7 | 2.303538 | 1.764033 | 3.054833 | 0.9897232 |
| age-1-4-5-6-7 | 2.346108 | 1.800368 | 3.068515 | 0.9896214 |
| age-2-3-4-5-6 | 2.503797 | 1.958902 | 3.299359 | 0.9879902 |
| age-2-3-4-5-7 | 2.464368 | 1.922569 | 3.215850 | 0.9886063 |
| age-2-3-4-6-7 | 2.456580 | 1.867086 | 3.240633 | 0.9884253 |
| age-2-3-5-6-7 | 2.359837 | 1.762057 | 3.105887 | 0.9893661 |
| age-2-4-5-6-7 | 2.379372 | 1.847797 | 3.111743 | 0.9893438 |
| age-3-4-5-6-7 | 2.447265 | 1.919073 | 3.199023 | 0.9887086 |
| age-1-2-3-4-5-6 | 2.238596 | 1.748163 | 2.947961 | 0.9904503 |
| age-1-2-3-4-5-7 | 2.208872 | 1.739636 | 2.875060 | 0.9909102 |
| age-1-2-3-4-6-7 | 2.241262 | 1.670777 | 2.936494 | 0.9905176 |
| age-1-2-3-5-6-7 | 2.104613 | 1.598536 | 2.801028 | 0.9913779 |
| age-1-2-4-5-6-7 | 2.156338 | 1.624312 | 2.839056 | 0.9911389 |
| age-1-3-4-5-6-7 | 2.166491 | 1.662423 | 2.845978 | 0.9911008 |
| age-2-3-4-5-6-7 | 2.214722 | 1.737097 | 2.882534 | 0.9908598 |
| age-1-2-3-4-5-6-7 | 1.991272 | 1.522724 | 2.624849 | 0.9924402 |

### ii) Age prediction with testing set

print(kable(t(tabGBM)))

|  | MAE | MeAE | RMSE | Cor |
| --- | --- | --- | --- | --- |
| age-1 | 6.390400 | 4.203610 | 8.955286 | 0.9104676 |
| age-2 | 6.817792 | 4.952942 | 9.334854 | 0.9032884 |
| age-3 | 7.884530 | 5.943945 | 10.499182 | 0.8744856 |
| age-4 | 7.959572 | 5.651598 | 10.449426 | 0.8750411 |
| age-5 | 6.211945 | 4.759553 | 8.417961 | 0.9212795 |
| age-6 | 4.928292 | 3.408164 | 7.222041 | 0.9421423 |
| age-7 | 6.182080 | 4.225068 | 8.549878 | 0.9182715 |
| age-1-2 | 6.154259 | 3.988306 | 8.610660 | 0.9173349 |
| age-1-3 | 6.006720 | 3.975433 | 8.502434 | 0.9200197 |
| age-1-4 | 5.919928 | 3.973371 | 8.411823 | 0.9219521 |
| age-1-5 | 5.478405 | 3.949316 | 7.700519 | 0.9348247 |
| age-1-6 | 4.980496 | 3.411283 | 7.056846 | 0.9446698 |
| age-1-7 | 5.282714 | 3.478833 | 7.454382 | 0.9388686 |
| age-2-3 | 6.359703 | 4.573625 | 8.871153 | 0.9134151 |
| age-2-4 | 6.162355 | 4.386809 | 8.673410 | 0.9173294 |
| age-2-5 | 5.412133 | 4.010949 | 7.673631 | 0.9359497 |
| age-2-6 | 4.908603 | 3.504927 | 6.965685 | 0.9462996 |
| age-2-7 | 5.297299 | 3.624559 | 7.511977 | 0.9384788 |
| age-3-4 | 7.094611 | 5.157658 | 9.465227 | 0.8992201 |
| age-3-5 | 5.624683 | 3.951405 | 7.821167 | 0.9323970 |
| age-3-6 | 4.863774 | 3.264190 | 7.049180 | 0.9448967 |
| age-3-7 | 5.854367 | 3.748867 | 8.261787 | 0.9238582 |
| age-4-5 | 5.508819 | 3.789851 | 7.660591 | 0.9358242 |
| age-4-6 | 4.905497 | 3.191432 | 7.018662 | 0.9453610 |
| age-4-7 | 5.891663 | 3.822227 | 8.277274 | 0.9233447 |
| age-5-6 | 4.570703 | 3.382342 | 6.656565 | 0.9511149 |
| age-5-7 | 5.172412 | 3.431543 | 7.260739 | 0.9420936 |
| age-6-7 | 4.760534 | 3.185453 | 6.917139 | 0.9470451 |
| age-1-2-3 | 5.915403 | 4.018928 | 8.375307 | 0.9224721 |
| age-1-2-4 | 5.774196 | 3.964525 | 8.251647 | 0.9252072 |
| age-1-2-5 | 5.385757 | 3.880132 | 7.654375 | 0.9354403 |
| age-1-2-6 | 4.993875 | 3.425055 | 7.051646 | 0.9447249 |
| age-1-2-7 | 5.219143 | 3.511366 | 7.393755 | 0.9401163 |
| age-1-3-4 | 5.787125 | 3.821510 | 8.268282 | 0.9246275 |
| age-1-3-5 | 5.388324 | 3.726319 | 7.607354 | 0.9364974 |
| age-1-3-6 | 4.902857 | 3.380613 | 7.039752 | 0.9449456 |
| age-1-3-7 | 5.236797 | 3.358541 | 7.493313 | 0.9382536 |
| age-1-4-5 | 5.193746 | 3.469338 | 7.444082 | 0.9396185 |
| age-1-4-6 | 4.870358 | 3.323364 | 6.993791 | 0.9457022 |
| age-1-4-7 | 5.276794 | 3.514413 | 7.460194 | 0.9389910 |
| age-1-5-6 | 4.752320 | 3.279547 | 6.756722 | 0.9494957 |
| age-1-5-7 | 4.965463 | 3.341458 | 7.004287 | 0.9459765 |
| age-1-6-7 | 4.755927 | 3.276578 | 6.837344 | 0.9481767 |
| age-2-3-4 | 6.070843 | 4.338694 | 8.606593 | 0.9185328 |
| age-2-3-5 | 5.249567 | 3.635998 | 7.518036 | 0.9377108 |
| age-2-3-6 | 4.849884 | 3.306107 | 6.898217 | 0.9472529 |
| age-2-3-7 | 5.199557 | 3.421734 | 7.459068 | 0.9391686 |
| age-2-4-5 | 5.200283 | 3.706656 | 7.406722 | 0.9399494 |
| age-2-4-6 | 4.822455 | 3.226483 | 6.917672 | 0.9470366 |
| age-2-4-7 | 5.278127 | 3.551670 | 7.475104 | 0.9389533 |
| age-2-5-6 | 4.530768 | 2.973097 | 6.586178 | 0.9521096 |
| age-2-5-7 | 4.826982 | 3.318525 | 6.930781 | 0.9480677 |
| age-2-6-7 | 4.639757 | 3.136984 | 6.748141 | 0.9498286 |
| age-3-4-5 | 5.454268 | 3.904386 | 7.615600 | 0.9366044 |
| age-3-4-6 | 4.869812 | 3.372818 | 6.987436 | 0.9459885 |
| age-3-4-7 | 5.824419 | 3.562954 | 8.192129 | 0.9253571 |
| age-3-5-6 | 4.528592 | 3.163852 | 6.627823 | 0.9514668 |
| age-3-5-7 | 5.125454 | 3.458544 | 7.282897 | 0.9416610 |
| age-3-6-7 | 4.732572 | 3.252772 | 6.838593 | 0.9481542 |
| age-4-5-6 | 4.494388 | 3.009961 | 6.538141 | 0.9528843 |
| age-4-5-7 | 5.088352 | 3.354188 | 7.143255 | 0.9439246 |
| age-4-6-7 | 4.790628 | 3.148265 | 6.912152 | 0.9471556 |
| age-5-6-7 | 4.500237 | 3.173759 | 6.489839 | 0.9535439 |
| age-1-2-3-4 | 5.692558 | 3.954728 | 8.182777 | 0.9261654 |
| age-1-2-3-5 | 5.278731 | 3.582038 | 7.560522 | 0.9370712 |
| age-1-2-3-6 | 4.878851 | 3.429753 | 6.968102 | 0.9460835 |
| age-1-2-3-7 | 5.135682 | 3.392273 | 7.367448 | 0.9401938 |
| age-1-2-4-5 | 5.122108 | 3.539202 | 7.449634 | 0.9390314 |
| age-1-2-4-6 | 4.888488 | 3.307268 | 7.001251 | 0.9455327 |
| age-1-2-4-7 | 5.206277 | 3.566868 | 7.381626 | 0.9404217 |
| age-1-2-5-6 | 4.715116 | 3.290821 | 6.728318 | 0.9498806 |
| age-1-2-5-7 | 4.896803 | 3.375582 | 6.984576 | 0.9467044 |
| age-1-2-6-7 | 4.736425 | 3.183365 | 6.823257 | 0.9483691 |
| age-1-3-4-5 | 5.210975 | 3.544952 | 7.475498 | 0.9385992 |
| age-1-3-4-6 | 4.844796 | 3.323834 | 6.989160 | 0.9457594 |
| age-1-3-4-7 | 5.193872 | 3.271858 | 7.466144 | 0.9386298 |
| age-1-3-5-6 | 4.685300 | 3.263301 | 6.729662 | 0.9498164 |
| age-1-3-5-7 | 4.934753 | 3.292934 | 7.009637 | 0.9460815 |
| age-1-3-6-7 | 4.716272 | 3.178921 | 6.824130 | 0.9483506 |
| age-1-4-5-6 | 4.630958 | 3.009021 | 6.670373 | 0.9510095 |
| age-1-4-5-7 | 4.913755 | 3.303652 | 6.969862 | 0.9469690 |
| age-1-4-6-7 | 4.727972 | 3.060158 | 6.815162 | 0.9485384 |
| age-1-5-6-7 | 4.600530 | 3.186576 | 6.570342 | 0.9523684 |
| age-2-3-4-5 | 5.172562 | 3.620364 | 7.333758 | 0.9408043 |
| age-2-3-4-6 | 4.809746 | 3.184986 | 6.882417 | 0.9475242 |
| age-2-3-4-7 | 5.218139 | 3.442629 | 7.479031 | 0.9388265 |
| age-2-3-5-6 | 4.516143 | 3.243006 | 6.552975 | 0.9525253 |
| age-2-3-5-7 | 4.785671 | 3.340572 | 6.885809 | 0.9480675 |
| age-2-3-6-7 | 4.593568 | 3.054782 | 6.688014 | 0.9504571 |
| age-2-4-5-6 | 4.472212 | 3.136506 | 6.507454 | 0.9534230 |
| age-2-4-5-7 | 4.782308 | 3.142242 | 6.887358 | 0.9482037 |
| age-2-4-6-7 | 4.659342 | 3.082398 | 6.756423 | 0.9497007 |
| age-2-5-6-7 | 4.443140 | 3.133932 | 6.424555 | 0.9547655 |
| age-3-4-5-6 | 4.512077 | 3.119790 | 6.553210 | 0.9526800 |
| age-3-4-5-7 | 5.046322 | 3.425806 | 7.154503 | 0.9433782 |
| age-3-4-6-7 | 4.751031 | 3.140073 | 6.871213 | 0.9477434 |
| age-3-5-6-7 | 4.472284 | 3.119901 | 6.488505 | 0.9535009 |
| age-4-5-6-7 | 4.494426 | 3.081183 | 6.490896 | 0.9536355 |
| age-1-2-3-4-5 | 5.134829 | 3.621671 | 7.421980 | 0.9395809 |
| age-1-2-3-4-6 | 4.859155 | 3.288749 | 6.979077 | 0.9459432 |
| age-1-2-3-4-7 | 5.127555 | 3.324134 | 7.367363 | 0.9405379 |
| age-1-2-3-5-6 | 4.673871 | 3.374139 | 6.692657 | 0.9504636 |
| age-1-2-3-5-7 | 4.874112 | 3.378314 | 6.945665 | 0.9469370 |
| age-1-2-3-6-7 | 4.690596 | 3.098720 | 6.786539 | 0.9489309 |
| age-1-2-4-5-6 | 4.619102 | 3.302272 | 6.638685 | 0.9512844 |
| age-1-2-4-5-7 | 4.859381 | 3.227670 | 6.964287 | 0.9470886 |
| age-1-2-4-6-7 | 4.743402 | 3.042032 | 6.835966 | 0.9481975 |
| age-1-2-5-6-7 | 4.528093 | 3.063661 | 6.497714 | 0.9534827 |
| age-1-3-4-5-6 | 4.644192 | 3.259971 | 6.681944 | 0.9505815 |
| age-1-3-4-5-7 | 4.910081 | 3.231080 | 6.993444 | 0.9465884 |
| age-1-3-4-6-7 | 4.706850 | 3.096041 | 6.805695 | 0.9486651 |
| age-1-3-5-6-7 | 4.571502 | 3.068346 | 6.537064 | 0.9527545 |
| age-1-4-5-6-7 | 4.587309 | 3.142736 | 6.571528 | 0.9524653 |
| age-2-3-4-5-6 | 4.507706 | 3.153201 | 6.515786 | 0.9530925 |
| age-2-3-4-5-7 | 4.780475 | 3.281526 | 6.855337 | 0.9483319 |
| age-2-3-4-6-7 | 4.607102 | 3.054772 | 6.699070 | 0.9503300 |
| age-2-3-5-6-7 | 4.420737 | 3.117159 | 6.387369 | 0.9550222 |
| age-2-4-5-6-7 | 4.425823 | 3.014072 | 6.411642 | 0.9547670 |
| age-3-4-5-6-7 | 4.477411 | 3.136781 | 6.462894 | 0.9538770 |
| age-1-2-3-4-5-6 | 4.589511 | 3.339790 | 6.652173 | 0.9510013 |
| age-1-2-3-4-5-7 | 4.834293 | 3.252753 | 6.919372 | 0.9475858 |
| age-1-2-3-4-6-7 | 4.668678 | 2.886058 | 6.760374 | 0.9493152 |
| age-1-2-3-5-6-7 | 4.551185 | 3.099787 | 6.516036 | 0.9530465 |
| age-1-2-4-5-6-7 | 4.517792 | 3.057231 | 6.486785 | 0.9535328 |
| age-1-3-4-5-6-7 | 4.574756 | 3.148044 | 6.551227 | 0.9527523 |
| age-2-3-4-5-6-7 | 4.433901 | 3.124688 | 6.408910 | 0.9546130 |
| age-1-2-3-4-5-6-7 | 4.547219 | 3.101357 | 6.489263 | 0.9534299 |

# II. Multiple Quadratic Regression (MQR) with Perl

For each age prediction obtained with all the possible combinations of DNA methylation values of the 7 CpGs and their square (16383 in total), the prediction accuracy was evaluated by the mean absolute deviation (MAD) and the root mean square error (RMSE). The correlation analyses were assessed using the Pearson R correlation coefficient.

#!/usr/bin/perl

use local::lib;
use Data::PowerSet 'powerset';

if ($#ARGV != 2) {
 print " usage: prg training_file testing_file variable_list_file\n";
 print " variable_list_file Tab-separated values (value1 = value2Explain ... explicative_values)\n";
 print "=================================\n";
 print " Fondation Jean DAUSSET - CEPH\n";
 print "=================================\n";
 print "Require installation of Perl Module PowerSet\n";
 print "cpanm Data::PowerSet\n";
 exit;
}

$training_file = $ARGV[0];
$testing_file = $ARGV[1];
$variable_list = $ARGV[2];

open (IN_VAL,"<$ARGV[2]") || die "Unable to open file corps.tmp\n";
while ($li = <IN_VAL>) {
 chomp $li;
 @tabVal = split(/\t/,$li);
 for ($i=1;$i<=$#tabVal;$i++) {
 $valin[$i-1] = $tabVal[$i],"\n";
 system("mkdir Uplet$i");
 }
 $title = "Index\tNbVal\tAdjusted-R-squared\tCOR-training_Set1\tRMSE1\tMAE1\tCOR-testing_Set2\tRMSE2\tMAE2\tModel\n";
 open (TMP,">log.LM.txt");
 print TMP $title;
 close(TMP);
 $cpt=0;
 my $powerset = powerset(@valin);
 for my $p (@$powerset) {
 $nbVal =0;
 print "#####\n";
 $cpt++;
 $nbVal = $#$p + 1;
 print "$cpt : $nbVal : @$p\n";
 $model = "mod".$cpt;
 $model2test = "lm(".$tabVal[0]."~";
 for ($i=0;$i<=$#$p-1;$i++) {
 $model2test = $model2test.$$p[$i]."+";
 }
 $model2test = $model2test.$$p[$#$p].",data=set1)";

 ### write R script file
 open (OUT_R,">tmp.R") || die "Unable to write\n";
 print OUT_R "\n";
 print OUT_R "require(stats)\n";
 print OUT_R "require(Metrics)\n";
 print OUT_R "set1 <-read.table(\"$training_file\",h=T)\n";
 print OUT_R "test <-read.table(\"$testing_file\",h=T)\n";
 print OUT_R $model." <- ".$model2test,"\n";
 print OUT_R "summary($model)\n";

 print OUT_R "predORI <- predict($model,set1)\n";
 print OUT_R "#pdf(\"predORI-cor.$model.pdf\")\n";
 print OUT_R "#plot(set1\$$tabVal[0],predORI,xlim=c(0,100),ylim=c(0,100))\n";
 print OUT_R "#abline(0,1,col=\"red\")\n";
 print OUT_R "#dev.off()\n";
 print OUT_R "cor(set1\$$tabVal[0],predORI)\n";
 print OUT_R "rmse(set1\$$tabVal[0],predORI)\n";
 print OUT_R "mae(set1\$$tabVal[0],predORI)\n";

 print OUT_R "predictions <- predict($model,test)\n";
 print OUT_R "#pdf(\"pred-cor.$model.pdf\")\n";
 print OUT_R "#plot(test\$$tabVal[0],predictions,xlim=c(0,100),ylim=c(0,100))\n";
 print OUT_R "#abline(0,1,col=\"red\")\n";
 print OUT_R "#dev.off()\n";
 print OUT_R "cor(test\$$tabVal[0],predictions)\n";
 print OUT_R "rmse(test\$$tabVal[0],predictions)\n";
 print OUT_R "mae(test\$$tabVal[0],predictions)\n";
 close(OUT_R);
 if ($nbVal >0) {
 system("Rscript tmp.R >res.$cpt.out");
 sleep(3);
 open (TMP,">tmp0");
 print TMP $log = $cpt."\t".$nbVal;
 close(TMP);
 system("cat res.$cpt.out | grep R-squared |awk '{print \$6}' >tmp1");
 system("cat res.$cpt.out | grep \"\\[1\" |awk '{print \$2}' >tmp2 ");
 transpose_matrice(tmp2,t,tmp3);
 system("cat tmp3");
 system("mv tmp.R Uplet$nbVal\/tmp.$cpt.R");
 system("mv res.$cpt.out Uplet$nbVal\/res.$cpt.txt");
 open (TMP,">tmp4");
 print TMP $model2test,"\n";
 close(TMP);
 system(" paste tmp0 tmp1 tmp3 tmp4 >>log.LM.txt");
 system("rm tmp0 tmp1 tmp2 tmp3 tmp4 ");

 } else {
 system("rm tmp.R");
 }
 }
}
close(IN_VAL);


# Function definition
sub transpose_matrice {
 my ($listA,$sep,$listB) = @_;
 $sepOut = "\t";
 $cptLI = 0;
 open (LISTA, "<$listA") || die " le fichier de selection $listA non lisible \n";
 open (LISTB, ">$listB") || die " le fichier de selection $listB non lisible \n";
 while ($li = <LISTA>) {
 chomp $li;
 $cptLI++;
 if ($sep eq "t") {
 @field_A = split(/\t/,$li);
 }
 if ($sep eq "s") {
 @field_A = split(/\s/,$li);
 }
 for ($i=0;$i<=$#field_A;$i++) {
 if ($assoTRANS{$i} eq "") {
 $assoTRANS{$i} = $field_A[$i];
 } else {
 $assoTRANS{$i} = $assoTRANS{$i}.$sepOut.$field_A[$i];
 }
 }
 }
 close(LISTA);
 foreach $cle (sort numeric keys %assoTRANS) {
 print LISTB $assoTRANS{$cle},"\n";
 }
 close(LISTB);
 %assoTRANS = ();
 @field_A = ();
}

sub numeric { $a <=> $b;}
